# In Vitro Antibacterial Efficacy of a New TiO_2_-Cu-Coated Titanium Surface for Biomedical Applications

**DOI:** 10.3390/nano15221742

**Published:** 2025-11-19

**Authors:** Mahalakshmi Pandian, Sacha Cavelier, Simone Guttau, Silvia Cometta, Joseph Fernando, Philipp Kobbe, Dietmar W. Hutmacher

**Affiliations:** 1 ARC Training Centre for Cell and Tissue Engineering Technologies, Queensland University of Technology, Kelvin Grove, QLD 4059, Australia; mahalakshmi.pandian@hdr.qut.edu.au (M.P.); dietmar.hutmacher@qut.edu.au (D.W.H.); 2 School of Mechanical, Medical and Process Engineering, Faculty of Engineering, Queensland University of Technology, Kelvin Grove, QLD 4059, Australia; silvia.comettaconde@qut.edu.au; 3 Max Planck Queensland Centre for the Materials Science of Extracellular Matrices, Queensland University of Technology, Kelvin Grove, QLD 4059, Australia; joseph.fernando@uq.edu.au; 4 Stryker Trauma GmbH, 24232 Schönkirchen, Germany; simone.guttau@stryker.com; 5 Department for Trauma and Reconstructive Surgery, University Hospital of the Martin Luther University Halle, Universitätsring 19/20, 06099 Halle (Saale), Germany; philipp.kobbe@bergmannstrost.de; 6 Centre for Biomedical Technologies, Queensland University of Technology, Kelvin Grove, QLD 4059, Australia; 7 Australian Research Council Training Centre for Multiscale 3D Imaging, Modelling, and Manufacturing, Queensland University of Technology, Kelvin Grove, QLD 4059, Australia; 8 Centre for Microscopy and Microanalysis, University of Queensland, Brisbane, QLD 4072, Australia; 9 Department for Trauma and Reconstructive Surgery, BG Klinikum Bergmannstrost Halle, 06112 Halle (Saale), Germany

**Keywords:** surgical site infection, copper, intramedullary nail, titanium implants, *S. aureus*, antibacterial surfaces, metallic coatings, electrochemical deposition

## Abstract

Despite advancements in surgical care, the management of surgical site infections (SSIs) associated with fracture-fixation devices is still a challenge after implant fixation, especially in open fractures. *Staphylococcus aureus* (*S. aureus*) is a common pathogen of SSIs and contaminates by penetrating the trauma itself (preoperatively) or during insertion of the fixation device (intraoperatively). A unique technology was developed to address this issue, consisting of an antibacterial surface obtained after depositing copper on a porous titanium oxide surface. This study aims to characterise and evaluate the in vitro bactericidal effect of this surface against *S. aureus*. Furthermore, the topography, elemental composition and other physicochemical properties of the copper coating were determined. In vitro assays have demonstrated a reduction of up to 5 log_10_ in the bacteria colonisation, and additional quantitative and qualitative methods further supported these observations. This study illustrates the antibacterial efficacy and killing mechanisms of the surface, therefore demonstrating its potential for minimising infection progression post-implantation in clinical scenarios and bringing important insights for the design of future in vivo evaluations.

## 1. Introduction

Surgical site infections (SSIs) are one of the most challenging complications associated with fractures and orthopaedic implants, especially in the case of open fractures, for which the infection rate can range from 10% to 50% [1,2]. In the case of open tibia fractures, the incidence rate of SSIs varies from 5 to 30% [3,4,5,6]. SSIs can lead to delayed fracture healing, functional loss, amputation and patient morbidity, and the hospitalisation length can increase to more than 7–11 days [7,8]. Additionally, fracture-related SSIs are associated with increased healthcare costs since treatments are up to 6.5 times more expensive for infected patients [9,10]. In the US, the financial burden of infected revisions was estimated to be $1.62 billion in 2020 [11]. SSIs are therefore the most dominant driver of total healthcare costs related to the surgical treatment of tibial fractures [12,13].

Most SSIs are reported to be associated with *Staphylococcus aureus* (*S. aureus*). The prevalence of these bacteria in SSIs has increased from 16.6% to 30.9% between 1992 and 2002 [14], reaching 37% in community hospitals [15]. Peri-implant *S. aureus* infections are challenging to treat, and conventional treatments require revision surgeries and long-term systemic antibiotic treatments. Current approaches consist of surgically removing the infected tissue and administering antibiotics, typically for 6–12 weeks [16,17,18]. Unlike other infections, implant-associated infections usually do not heal spontaneously, and prolonged antibiotic therapy can lead to the development of antibiotic resistance [19]. Antibiotic resistance is a growing concern as *S. aureus* tends to evolve into methicillin-resistant *S. aureus*, for which the number of isolates has risen from 9.2% to 49.3% [14]. In addition, the invasiveness and functional complications of surgical revisions have contributed to the consideration of antimicrobial surfaces as an alternative therapy to circumvent the limitations associated with systemic antibiotic treatments [20,21].

Antimicrobial coatings on the surface of titanium (Ti) implants have two goals: (1) prevent infection and (2) enhance fracture repair [22]. Antibiotic-coated nails for orthopaedic trauma surgery have been widely investigated [23,24,25,26] and enable the local release of drugs, resulting in bacterial reduction and improvements in bone healing [27]. However, the growing emergence of antibiotic-resistant bacteria may limit the efficacy of antibiotic coatings. Antiseptic coatings are presented as an alternative due to their broad-spectrum antibacterial, antiviral, and antifungal properties [28,29,30]. For instance, fluoride-doped TiO_2_ coating on Ti implants [31], titanate–iodine coating [32] or iodine-coated Ti implants [33] exhibited antimicrobial activity against various bacteria and fungi. However, antiseptic coatings can interfere with the implant’s integration into surrounding tissue and cause allergic reactions [34], and their effectiveness diminishes over time, adding complexity and cost [35]. Nanostructured surfaces have also emerged as a key method for eradicating bacteria by controlling adhesion or friction, or even by inducing structural damage and oxidative stress. These surfaces encompass a diverse range of textures, including nanoripples, nanopillars, nanotubes, nanowires, nanoprotrusions, and nanoparticles [36,37,38]. Nanostructured surfaces also include superhydrophobic surfaces, which deter bacterial colonisation due to their water-repellent nature [39]. However, their lack of durability and resistance to corrosion limits their efficacy [40], and they fail to combine both efficiency and economic feasibility, as the fabrication techniques can be too expensive at a large scale [36].

Finally, seventeen metal elements are known today to exhibit antimicrobial activity [41,42], with silver (Ag), zinc (Zn), and copper (Cu) being the most commonly used [43]. They possess a broad spectrum of antibacterial properties that assist in the prevention of bacterial implant contamination, particularly in surgical equipment [44]. Bactericidal mechanisms primarily consist of contact-killing, where bacteria are killed upon contact with the metallic elements at the surface [45,46]. For instance, Ag-coated Ti implants can significantly reduce bacterial colonisation by disrupting the cell wall and affecting cellular metabolism [45]. Zn and its oxide, ZnO, exhibit antimicrobial properties against a wide spectrum of microorganisms, including drug-resistant bacteria [47], and antiviral properties, as it disrupts viral protease and RNA or DNA polymerase functions, as well as viral attachment [48]. However, Ag and Zn can be cytotoxic, inhibiting both cell growth and tissue healing around the implant site [49,50]. In some cases, bacterial strains can develop resistance to Ag over time, reducing its effectiveness in preventing infection [49]. Mg has also been studied as an antibacterial coating; however, the small impurity in the Mg matrix is responsible for rapid degradation and corrosion, which is problematic for orthopaedic applications [51]. Alternatively, MgO can be used to increase both corrosion resistance and antibacterial effect, but the exact killing mechanisms have not been elucidated yet [52].

Cu, the most widely used bactericidal metallic coating, is a trace element in the human body, and therefore exhibits a high cytocompatibility and lower levels of toxicity, alleviating the limitations previously mentioned [53,54]. Unlike other metals, Cu has a lower likelihood of developing resistance to bacteria [53,55]. It is already employed in a variety of products, including door handles, handrails, and textiles, as well as in biomedical applications and healthcare settings, such as intrauterine devices, bed linens, patient gowns, and wound dressings [56]. Indeed, Cu exhibits proficient contact-killing and inhibits bacterial growth [53]. The release of Cu ions, specifically Cu^+^ and Cu^2+^, may cause damage to the bacterial membrane, thereby decreasing cell invasion and triggering an oxidative stress response that leads to lipid peroxidation, membrane disruption, protein impairment, and cell death [41,54,55]. As a result, essential cellular components leak out, contributing to bacterial death [54]. Additionally, Cu ions can penetrate the bacteria, causing DNA damage and disrupting vital cellular processes such as replication and transcription [57]. Overall, the multifaceted antibacterial mechanisms of Cu target various cellular components and processes, making it an effective antimicrobial agent [54]. Cu alloys have demonstrated effectiveness against a wide spectrum of pathogens, rapidly eradicating 107 *S. aureus* colony forming units (CFU) per ml within 10 min, *Acinetobacter* within 240 min, or *E. coli* within 350 min, therefore reducing the bacterial load by a factor of 105 [43]. Other studies on Cu coatings and Cu alloys have demonstrated significant in vitro reductions in *S. aureus*, by factors ranging from 103 to 1010 after a few hours [55,58,59,60,61,62], with greater efficacy observed in moist environments [58].

A novel electrochemical coating process was developed to deposit metallic Cu at the surface of medical-grade Ti implants. Such a technology could have direct biomedical applications, such as intramedullary nails with bactericidal coatings for bone fracture repairs. Briefly, the fabrication process consists of electrochemical deposition of Cu into a Ti oxide layer, followed by glass bead blasting. Compared to other fabrication techniques, the resulting surface (where the copper is embedded in the anodization layer) does not impact the stability of the implant and is not abrasive, and the topology of the surface upon Cu release is similar to that of anodized Ti implants. More generally, Cu coatings generated via electrochemical deposition, or *electrodeposition*, exhibit columnar and nanograined structures that promote antimicrobial activity. This fabrication technique is also advantageous because of its low costs, the uniform distribution of the coating, and its easy application onto metal surfaces, even on highly complex objects [63]. Electrodeposition techniques were successfully used to create metallic coatings with antibacterial properties on a variety of surfaces, as summarised in Table 1. This table provides an overview of electrodeposited antibacterial metallic coatings from literature, showing that Cu, Ag and Zn are the most popular coatings. Overall, better antibacterial performances can be attributed to Cu coatings, but the efficacy of each coatings varies significantly across studies, due to variations in the designs of the coating or in the testing protocols. It is therefore essential to individually test novel antibacterial coatings. The evaluation of the physico-chemical and antimicrobial properties of electrodeposited Cu coatings with glass bead blasting has only just been initiated in recent work from Giraldo-Osorno et al. [64]. We propose an extended characterisation of the surface using additional techniques, along with a reproduction of the in vitro antibacterial evaluation against *S. aureus*, but with extra timepoints and higher concentrations. These additional data will be central to the design of future in vivo evaluations. The hypothesis that the novel Cu coating on Ti is capable of reducing bacterial colonisation over time in vitro, at a higher rate than anodized Ti without Cu coating after inoculation of the strain, will be tested. If successful, the outcomes will pave the way for future in vivo evaluations in large animal infection models and will accelerate the regulatory approval of Cu-coated orthopaedic implants.

## 2. Methods

### 2.1. Materials

**Figure 1:**
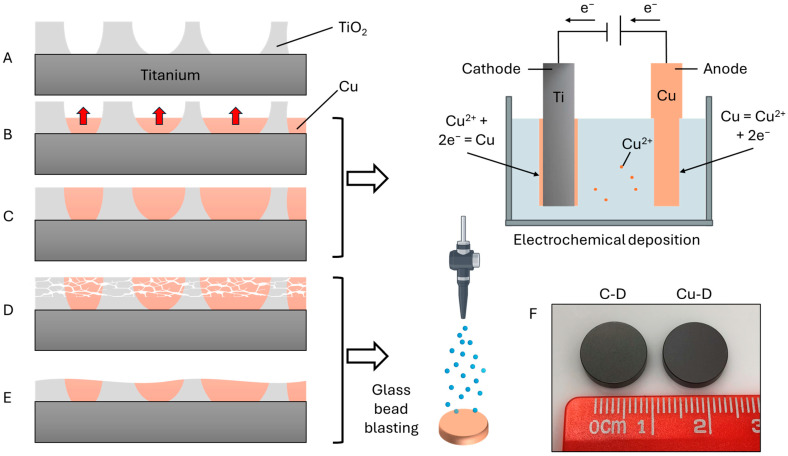
Schematic diagram of the fabrication process: (**A**) A porous TiO_2_ layer was formed. (**B**,**C**) Cu was deposited into the pores by electrochemical deposition. (**D**,**E**) Glass bead blasting was used to remove the excess Cu and friable TiO_2_. (**F**) View of the C-D and Cu-D discs.

Discs made of Ti6Al4V-ELI with a diameter of 12 mm and a height of 3 mm were manufactured by Stryker Trauma GmbH, Schönkirchen, Germany, following the same fabrication protocol as previous studies [64]. For the control Ti discs (C-D group), a porous Ti oxide (TiO_2_) layer was formed by anodization in sodium hydroxide solution. The samples were then glass-bead-blasted (⌀ 40–70 μm), cleaned, sterile packed, and gamma-sterilised at 25–50 kGy in accordance with ISO 11137 [84]. For the Cu-coated discs (Cu-D), the Cu coating was prepared according to European patent EP 2 204 199 B1. The samples were also anodized in sodium hydroxide solution (Figure 1A), followed by the electrodeposition of Cu into the pores (Figure 1B,C). The excess of Cu and TiO_2_ was removed through the glass bead (⌀ 40–70 μm) blasting technique (Figure 1D,E). The Cu-coated discs (Cu-D group) were then cleaned and sterile packed using gamma-sterilisation (25–50 kGy) for further experimentation. Anodizing, as well as the antibacterial Cu coating, were developed and carried out by DOT GmbH (DOT GmbH, Rostock, Germany). Final discs are displayed in Figure 1F, illustrating that a slightly darker colour was observed on the Cu-D discs compared to the C-D discs.

Finally, *S. aureus* subsp. *aureus Rosenbach* ATCC 6538 (ATCC^®^ 6538-MINI-PACK™, ATCC, Manassas, VA, USA) was used in this study.

### 2.2. Scanning Electron Microscopy (SEM) and Energy Dispersive Spectroscopy (SEM-EDS)

The microstructure of the three C-D and three Cu-D samples was analysed using SEM (MIRA3 FEG-SEM, TESCAN, Brno, Czech Republic). The samples did not receive additional metallic coating since they are conductive materials. Plasma cleaning (Evactron^®^ 25Z & Soft Clean, Zephyr, Sydney, Australia) was performed for 5 min before imaging to remove impurities. High-resolution images were captured at 5 kV voltage and 8 W/m^2^ beam intensity in backscattered detector mode, at a working distance of 8 mm. Image analysis was conducted with the software ImageJ, version 1.54i (https://imagej.net/ij/, accessed on 15 April 2024), employing binary image analysis and colour histogram. The chemical composition of the samples was investigated by EDS (Phenom XL G2 Desktop SEM, ThermoFisher Scientific, Brisbane, Australia) immediately after imaging with the SEM. For this purpose, the SEM was operated at a magnification of 1000×, with a voltage of 15 kV and a working distance of 10 mm, and one sample per group was analysed in six different locations.

Morphological changes in *S. aureus* on two Cu-D and two C-D samples at different incubation times (4 h, 24 h, 72 h, details in Section 2.9) were also observed using the SEM instrument, with the same settings. For this purpose, after fixing the bacteria in 4% (*v*/*v*) paraformaldehyde (PFA) solution, the samples were dehydrated using ethanol baths with progressively increasing concentrations (10 min in 20 to 100% ethanol, increment of 10% each time). The samples were critical point dried and sputter-coated with a 2 nm platinum conductive layer.

### 2.3. Inductively Coupled Plasma Optical Emission Spectroscopy (ICP-OES)

C-D and Cu-D samples were transferred and accurately weighed into 50 mL polypropylene digestion tubes before the addition of 5 mL of a 1:1 HNO_3_:H_2_O mixture prepared from twice sub-boiling distilled HNO_3_ and deionized water. The samples and solutions were then briefly agitated on an ultrasonic bath for 60 s before being placed on a graphite heating block set at 110 °C for 45 min twice. Residual liquids were transferred into aliquots for analysis by ICP-OES using a combination of axial and radial viewing modes. This was achieved using an Optima 8300 ICP-OES (Perkin Elmer, Waltham, MA, USA) fitted with an ESI SC-4DX autosampler and PrepFAST 2 sample handling unit for online internal standardisation, auto-dilution of samples and calibration standards.

### 2.4. Xray Photoelectron Spectroscopy (XPS)

The elemental composition of the surface of three C-D and three Cu-D samples was analysed using XPS (AXIS Ultra, Kratos Analytical, Manchester, UK). Initial survey spectra were obtained at a pass energy of 160 eV to provide an overview of the surface elements. Subsequently, high-resolution scans of specific elements (O 1s, C 1s, Ti 2p, Cu 2p, Al 2p, and Si 2p) were conducted at a pass energy of 20 eV, allowing for a more detailed analysis of their chemical states and concentrations. Atomic concentrations of the elements present on the surface were calculated from the survey spectra using the CasaXPS software version 2.3.19.

### 2.5. Ultraviolet Photoelectron Spectroscopy (UPS)

UPS was conducted to estimate the surface potential of one C-D and one Cu-D samples, on two locations for each sample. UPS measurements were performed using an Axis Supra+ (Kratos, Manchester, UK) instrument equipped with a He (I) ultraviolet radiation source. All samples were lightly cleaned using a Minibeam 6 gas cluster ion source operated at 10 keV Ar_2000_^+^ cluster mode for 30 s prior to acquisition. To ensure accurate energy calibration, a gold reference sample was measured under the same experimental conditions as the samples. The secondary electron cutoff (SECO) of gold was used to calibrate the energy scale, assuming a work function of 5.1 eV. A sample bias of −9 V was applied to accurately determine the secondary electron cutoff.

### 2.6. Profilometry

The surface roughness and micro-topography were examined by a profilometer (Dektak XTL, Bruker, Billerica, MA, USA) which measures roughness parameters according to ISO 21920-2:2021 standard [85]. *R_a_* (roughness calculated by arithmetical mean deviation) was calculated upon acquisition of data for three C-D and three Cu-D samples following the formula:

(1) $$R_{a}\;=\;\frac{1}{n}\sum_{i=0}^{n}\left|\gamma_{i}\right|$$

where *n* denotes the number of data points within the sampling length and *γ_i_* represents the difference between the actual height at a specific data point and the mean height of the sampling length.

### 2.7. Sessile Dynamic Contact Angle

The surface wettability was assessed using the sessile drop technique with a contact angle metre (ThetaFlex Drop Shape Analyser, Biolin, Gothenburg, Sweden). The contact angle of the surface of three C-D and three Cu-D samples was determined using 5 µL droplets of distilled water at room temperature. The image of the water droplet spreading and retraction was captured for 10 s after delivery. High-speed cameras and contact angle goniometers with video capturing capabilities were used to record the dynamic behaviour. The images were used to automatically measure the contact angle over time through the instrumental software.

### 2.8. Cu Release Profile

The assessment of the Cu release profile of the coating was adapted from a protocol by Giraldo-Osorno et al. [64] who determined the release kinetics up to 7 days. In the present study, the Cu release experiment was extended to 14 days. The Cu leaching behaviour of three Cu-D samples was assessed in 1 mL of Dulbecco’s modified Eagle medium with foetal bovine serum at 37 °C to mimic the body fluid. The Cu^2+^ release was measured using ICP-OES (Optima 8300, Perkin-Elmer, Waltham, MA, USA) according to the ISO 10993-12:2021 standard [86]. At each timepoint, the media was replaced and transferred into polypropylene digestion tubes along with a 1:1 HNO_3_:H_2_O mixture. After ultrasonic agitation and heating at 110 °C for 45 min, followed by a subsequent 60 min heating with additional water, the samples were cooled, decanted, and transferred for analysis to measure the cumulative Cu^2+^ concentration to evaluate their release behaviour.

### 2.9. Optical Densitometry

In this study, the target bacterial concentration of 1–5 × 10^8^ CFU/mL, corresponding to an optical density (OD_600_) of 0.06. *S. aureus,* was grown overnight on Mueller–Hinton agar plates, then resuspended in Tryptone Soya Broth (TSB) and measured at 600 nm using a Beckman DU 800 Spectrophotometer (Beckman Coulter, Brea, CA, USA). The actual bacterial concentration in the broth was determined by serial dilution, as detailed in the next paragraph.

### 2.10. Antibacterial Activity Assay Measured by CFU Count

All CFU count analyses in this study were performed by the serial dilution technique. Serial dilutions of each sample were plated on Mueller Hinton agar and incubated overnight. The count of individual colonies was facilitated by imaging via ChemiDoc Imaging System (Bio-Rad, Hercules, CA, USA).

For the antibacterial activity assay, a protocol was adapted from similar studies on different surfaces [87,88,89,90], and from Giraldo-Osorno et al. [64], who evaluated the same Cu coating. Here, the in vitro evaluation was extended by using an additional timepoint (72 h) and a higher bacterial inoculum. *S. aureus* bacteria were defrosted and streaked onto agar plates to produce fresh cultures upon overnight incubation. To prepare a 0.5 McFarland standard suspension (corresponding to a turbidity of 0.06 OD_600_ value at 600 nm, equivalent to 1–5 × 10^8^ CFU/mL), single *S. aureus* colonies from the streak plate were suspended in TSB. OD_600_ measured the initial concentration at t = 0 h, and a more accurate measurement of the number of CFU was performed later by the serial dilution technique. 1 mL of bacterial suspension was added to twenty-four C-D samples (positive control group) and twenty-four Cu-D samples (experimental group), which were incubated at intervals of 4 h, 24 h, and 72 h. In addition, twenty-four C-D samples received sterile TSB (negative control group) and were incubated at the same intervals, to detect potential cross-contamination. At 4 h and 24 h, six discs of each group (negative control, positive control, and experimental) were washed twice with 500 µL of sterile PBS and subsequently sonicated twice for 15 min in 2 mL of sterile PBS to recover the adhered bacteria. The CFU counting was achieved by serial dilution of the recovered bacterial cultures on Mueller-Hinton agar plates, upon overnight incubation. The remaining discs (twelve for each group) were incubated for 72 h, but half of the discs had their media changed at 24 h and 48 h, to eliminate the accumulation of Cu released from the Cu-D samples in the media, as well as to provide fresh nutrients for bacterial growth. Table 2 summarises the different groups evaluated in this assay. After 72 h, the samples underwent the same protocol as the other timepoints (washed twice in 500 µL of sterile PBS, sonicated twice for 15 min in 2 mL of sterile PBS and serially diluted and plated on Mueller-Hinton agar).

### 2.11. Presto Blue Assay

Six samples each of C-D and Cu-D were placed into the wells of a 24-well plate. Each sample was then inoculated with 1 mL of *S. aureus* suspension (1–5 × 10^8^ CFU/mL) in Luria–Bertani (LB) broth and incubated at 37 °C for different time intervals (4 h, 24 h, 72 h). After each timepoint, the respective C-D and Cu-D samples underwent a double PBS wash, followed by transfer to a new well plate. A 1X solution of Presto Blue reagent was diluted in LB broth (1:10 dilution) and 500 µL were added to each C-D and Cu-D containing well and incubated at 37 °C for 1 h. Afterward, elutes were transferred to a 96-well plate in triplicate, and fluorescence was quantified using a plate reader (excitation: 560 nm, emission: 590 nm). The remaining Presto Blue and LB broth solutions were discarded, and the disc-containing wells were washed twice with LB broth before being refilled with fresh LB broth. The plate was re-incubated at 37 °C until the next timepoint. The procedure was repeated for each timepoint.

### 2.12. Live-Dead Staining for Confocal Imaging

*S. aureus* stock solution was cultured in LB broth for 24 h, and the concentration was adjusted to 0.5 McFarland, as previously described. The suspension containing approximately 1–5 × 10^8^ CFU/mL was added to Cu-D samples placed in a 48-well plate and incubated at 37 °C for 4 h, 24 h or 72 h. Post-incubation, the discs were washed, stained for 15 min with Syto 9 and propidium iodide (PI) dyes, and fixed with 4% (*v*/*v*) PFA. Live and dead bacteria were detected by fluorescent imaging with a confocal microscope (Leica TCS SP5, Leica Microsystems, Wetzlar, Germany). The sequence was repeated for each timepoint (4 h, 24 h and 72 h).

### 2.13. Statistical Methods

All experimental data were expressed as average ± standard deviation (SD). Statistical comparisons between groups were performed by calculating *p*-values using a two-tailed, homoscedastic Student’s t-test for pairwise comparisons. Statistical significance was defined as *p* < 0.05.

## 3. Results and Discussion

### 3.1. SEM Observation and EDS Elemental Analysis

**Figure 2:**
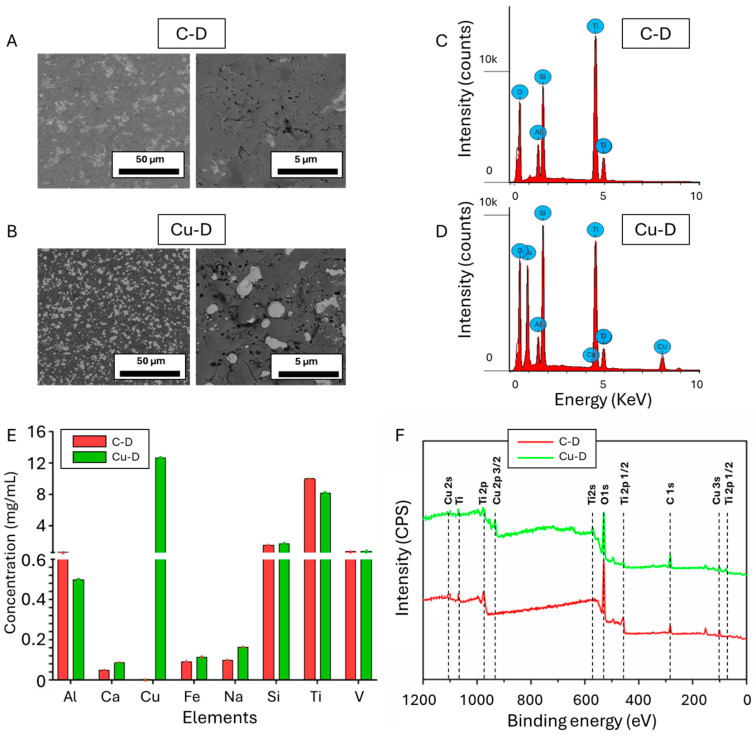
SEM imaging of (**A**) C-D sample and (**B**) Cu-D sample, at two different magnifications. Intensity spectra of (**C**) C-D sample and (**D**) Cu-D sample. (**E**) ICP-OES concentration analysis. (**F**) Survey spectrum of the XPS analysis for the two groups.

SEM and EDS elemental analysis were initially used to assess the morphology and elemental composition of the C-D and Cu-D surfaces. Areas with brighter contrast were observed on the samples from the Cu-D group, as shown in Figure 2B, but not in the C-D group, as shown in Figure 2A. Those contrasted areas can be attributed to the presence of Cu, because of the difference in conductivity between Cu and Ti. Cu particles were randomly and densely arranged onto the Ti surface, exhibiting a range of sizes between 0.5 and 5 µm. Upon analysis of the corresponding binary images using ImageJ, the Cu coverage of the surface was calculated to be 31.0 ± 5.0%. Similar observations were reported by Giraldo-Osorno et al. [64] who characterised the same coating technology and described the surface as island-like Cu deposits.

Figure 2C and 2D show the energy-versus-intensity spectra obtained by EDS analysis for the C-D and Cu-D samples, respectively. The intensity peaks of the C-D samples’ spectra were characteristic of the presence of oxygen (O), aluminium (Al), silicon (Si), Ti, and vanadium (V). The presence of Ti, Al, and V in the C-D samples, and the proportion of these elements, given by Supplemental Table S1, followed the expected composition of medical grade Ti-4Al-6V alloy [91,92]. A similar composition was revealed in the Cu-D samples, but with Cu at a concentration of 23.1 w% (Supplemental Table S1). The presence of oxygen in high quantities was attributed to the oxide forms of Ti, TiO_2_, specifically on the surface that were deposited during the fabrication process. The decrease in the elemental composition when comparing Cu-D to C-D (e.g., 47.0 w% vs. 31.2 w% for Ti, 35.7 w% vs. 27.4 w% for O, or 4.9 w% vs. 3.6 w% for Al) is the result of Cu covering the surface instead of Ti-4Al-6V alloy or TiO_2_. Finally, the presence of Si was due to the glass bead blasting procedure, during which ceramics, including SiO_2_, were projected at the surface. The glass bead blasting process on Ti surfaces can indeed lead to the contamination of the surfaces by elements related to the composition of the beads, such as O, Si, sodium (Na), magnesium (Mg), and calcium (Ca) [92,93].

### 3.2. Cu Surface Elemental Concentration Measured Through Inductively Coupled Plasma Optical Emission Spectrometry (ICP-OES)

Figure 2E displays the results of the ICP-OES analysis, which was also used to quantify the elemental composition of C-D and Cu-D surfaces. The detected elements were similar to those detected with EDS, indicating the presence of Ti, Al, V, Si, and Cu in the Cu-D samples only. Additionally, as depicted in Supplemental Table S1, the higher sensitivity of the technique enabled the detection of additional elements such as Ca and Na, at low concentrations. This can be attributed to the glass bead blasting processing, which involves SiO_2_, Na_2_O, CaO, MgO, and Al_2_O_3_ [92,93]. Iron (Fe) was also detected in low concentrations, as part of the composition of Ti-6Al-4V alloy [94]. Due to the different nature of the analysis, which consists of the dissolution of a thin layer at the surface of the samples, the proportion of the elements differed from the EDS analysis. For instance, the concentration of Cu detected with ICP-OES was lower, as a thicker layer of the TiO_2_-Cu coating may have been dissolved during the analysis.

### 3.3. X-Ray Photoelectron Spectroscopy (XPS)

Finally, the elemental composition of C-D and Cu-D samples was also investigated through XPS, and the survey spectra are shown in Figure 2F. Carbon, O, Ti, Si, and Na were detected in both groups, and Cu, and Al in Cu-D only. The presence of carbon is the result of residual organic contamination that could have persisted despite plasma cleaning. High-resolution spectra taken in the Ti 2p regions on surface of the C-D and Cu-D samples (Figure S1 in the Supplemental Data) evidenced a peak characteristic of Ti^4+^ (TiO_2_) at 459 eV, which dominated with a lower oxidation state fitted for Ti^3+^ at 461 and 464 eV. The Ti 2p spectrum depicts the typical binding energies of Ti dioxide (TiO_2_) [95], thus demonstrating the presence of TiO_2_. The Ti^3+^ suggests that this oxidation state exists throughout the oxide layer. Similarly, to C-D, Cu-D exhibited the Ti^4+^ without any Ti^3+^ peak, indicating a reduction in the oxidation state of Ti in the TiO_2_ form. This reduction was attributed to the presence of Cu in its oxidised form, with the impact of Cu deposition being noticeable in Cu-D [95]. In contrast, in the Cu high-resolution spectra, C-D samples did not show any peaks for Cu and Cu-D samples showed peaks at 935 and 955 eV, thus evidencing the presence of Cu^2+^, Cu^+^ or Cu species (Figure S1 in Supplemental Data). Moreover, the peaks of the Cu 2p at 935 and 955 eV can be attributed to the formation of Cu^2+^ on the surface [96].

The atomic concentrations were calculated and are indicated in Supplemental Table S2. The alterations in the elemental makeup of oxygen, Ti, and Cu suggest that Cu has been successfully bonded to the surface of the Ti disc. The drop in Ti (1.5 versus 5.5%) and O (32.9 versus 50.5%) when comparing Cu-D to C-D indicates a reduction in elemental composition due to Cu covering the surface instead of Ti and its oxide form, TiO_2_. The presence of Al was primarily due to the composition of the bulk material (Ti6Al4V), while Si and Na were likely due to the glass beads used in the sandblasting technique during sample preparation.

### 3.4. Ultraviolet Photoelectron Spectroscopy (UPS)

The surface work function *ϕ* of the sample could be calculated using the following equation [97]:

(2) ∅ = hυ − SECO

where hυ is the photon energy of the UV excitation source, e.g., 21.2 eV in this analysis. The SECO was determined from the high binding energy side of the UPS spectrum, and the valance band maximum (VBM) of the sample could be calculated from the lower binding energy region of the spectrum. The surface band bending potential (*V_bb_*) was estimated by the work function difference between the surface and the bulk [98,99].

The SECO, determined from the high binding energy side of the UPS spectrum, as shown in Supplemental Figure S2A, was found to be 16.5 eV. Hence, the work function of the C-D sample, based on Equation (2), was 4.7 eV. The VBM of the C-D sample was then calculated from the lower binding energy region of the spectrum also shown in Supplemental Figure S2A. The VBM was determined to be 3.1 eV which is characteristic of an oxidised Ti surface [100]. XPS measurements also confirmed the presence of an oxidised Ti surface (as shown in previous section). The bulk work function of TiO_2_ was taken as 4.4 eV from literature [98]. Finally, based on the measured work function and the bulk work function of TiO_2_, the surface band bending energy for the C-D sample was estimated as +0.3 eV, which indicates an upward band bending and a positive surface potential for a n-type semiconductor such as TiO_2_. Supplemental Figure S2B shows the UPS spectrum of the Cu-D sample. A work function of 4.4 eV and a VBM of 0.9 eV was calculated, which can be expected for an oxidised Cu surface [101]. XPS measurements also further confirmed the presence of an oxidised Cu surface on the Cu-D sample. The bulk work function of Cu_2_O was taken as 4.8 eV [102], thereby giving a surface band bending energy of −0.4 eV, which indicates a downward band bending and a negative surface potential for a *p*-type semiconductor such as Cu_2_O. Previous studies have shown that a surface negative potential can inhibit bacterial adhesion and biofilm formation [103]. The changes in surface potential of the Cu-D group could therefore have affected bacterial adhesion, as discussed in Section 3.6.

### 3.5. Surface Profilometer

**Figure 3:**
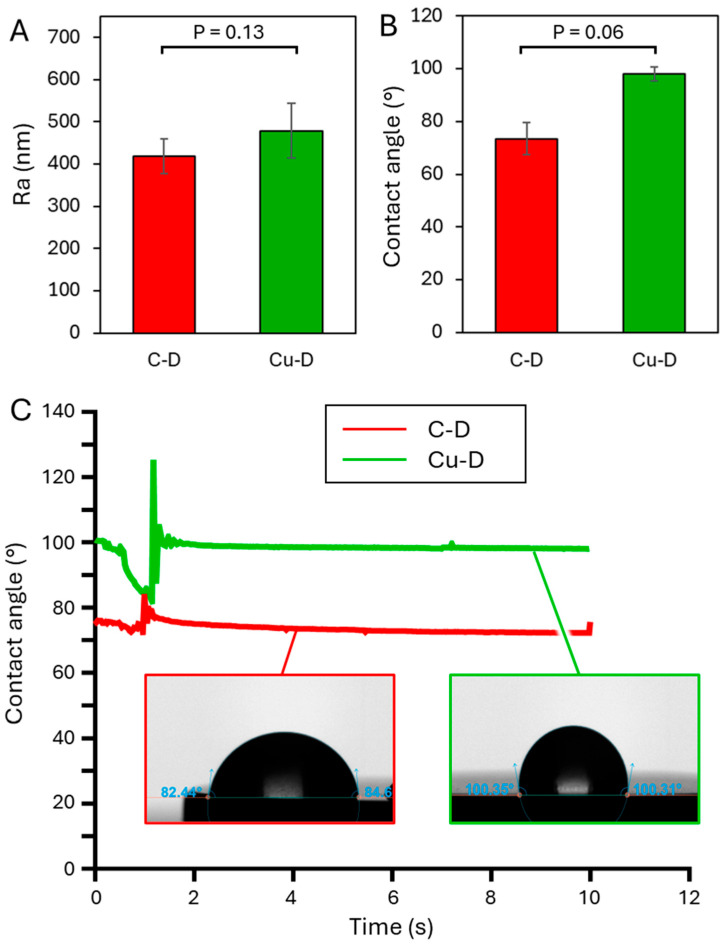
(**A**) Surface roughness and (**B**) contact angle measurements for the C-D and Cu-D groups. (**C**) Evolution of contact angle over 12 s.

The surface profilometry results of the C-D and Cu-D groups are shown in Figure 3A. The average surface roughness *R_a_* of C-D and Cu-D samples was 418.7 ± 41.7 nm and 479.0 ± 64.5 nm, respectively. This increase in roughness for the Cu-coated surface was also observed in the study by Giraldo-Osorno et al. [64]. Such a difference in roughness can be attributed to the presence of Cu at the surface of Cu-D samples. Indeed, the antibacterial coating consists of a combination of a hard and stiff porous TiO_2_ ceramic layer and more ductile Cu agglomerates. Because of the dissimilar mechanical properties of the two materials, it can be hypothesised that Cu and TiO_2_ were not removed in the same proportions during the blasting process, thus revealing a more irregular topography on the Cu-D sample surface. For instance, the Vickers hardnesses of Ti and TiO_2_ are 352 Hv [104] and 228 Hv [102], respectively, while Cu has a hardness of 107 Hv [105].

### 3.6. Contact Angle–Dynamic Mode

The contact angle measurements were evaluated through the sessile drop technique to reveal the wetting characteristics of the two different sample groups C-D and Cu-D (Figure 3B,C). For C-D samples, the contact angle was 73.5 ± 6.0° (compared to 70.23° for Giraldo-Osorno et al. [64]), indicating hydrophilicity. For Cu-D samples, however, a contact angle of 98.0 ± 2.5° (compared to 105.05° for Giraldo-Osorno et al.) was measured, indicating hydrophobicity. Similarly to roughness, this increase in the contact angle can be attributed to the glass blasting process, during which heterogeneous surfaces and micropores are created. Such micropores act as air cavities, which prevent spontaneous wetting [106]. These changes in the surface properties of the Cu-D group could have affected bacterial adhesion. Indeed, a study on Ti6Al4V demonstrated that *S. aureus* bacterial adhesion could be prevented on more hydrophilic surfaces [107]. However, it is unclear whether the improved hydrophobicity observed in the Cu-D samples could reduce or contribute to the antibacterial efficacy. Dong et al. [108] studied Ag metallic coatings and showed that the antibacterial effect associated with wetting properties was negligible compared to the impact due to the presence of Ag. On the other hand, improved antibacterial behaviour of Cu surfaces is usually attributed to high hydrophilicity that reduces bacterial adhesion on the surface, as observed with hydrophilic Cu coatings tested against *S. aureus* [109]. Notably, Nie et al. [61] prepared electrodeposited continuous Cu coatings and Cu-dotted coatings on stainless steel (covering 15–20% of the surface) with superior hydrophilicity. The Cu-dotted coatings exhibited better antibacterial properties compared to samples with continuous Cu coatings. This effect was attributed to an enhanced contact killing mechanism. Notably, the increased hydrophilicity observed in this study differs from the results in our study, which is attributed to the absence of glass bead blasting in the fabrication process and a distinct surface morphology.

### 3.7. Cu Release Kinetics via ICP-OES

**Figure 4:**
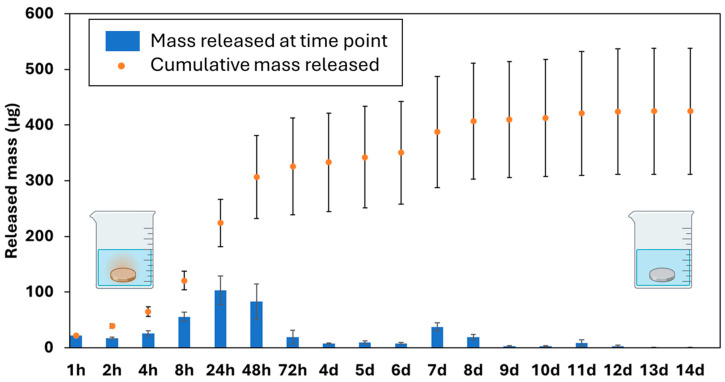
Cu release kinetics for a single Cu-D sample.

The cumulative release and release profile for Cu-D samples are shown in Figure 4. The release profile can be divided into two stages: an immediate burst release until day 3, followed by a low-rate release that persists until day 12. After day 13, no trace of Cu^2+^ was detected in the leachate, indicating that the Ti surface had been cleared of Cu. During the burst release, 224 ± 43 µg were released after 24 h, and 326 ± 87 µg after 72 h, which correspond to 53% and 76% of the cumulative release after 14 days (425 ± 113 µg). The surface was therefore capable of releasing 0.96 µg/mm^2^ within the first 3 days after release. Such release rate could probably not reach cytotoxic levels for cells in an in vivo context, since lowest Cu cytotoxic levels reported in literature stands at 46 μg/mL [110]. For instance, an in vivo study on a rat model used Ti plates Cu-coated with the same technology and with 70 mm^2^ of total surface exposed (in comparison, Cu-D samples have a surface of 339 mm^2^), and the observed tissue reactions were of minor importance compared to the presence of implant themselves; however, a lower concentration in the Cu coating (0.2 µg/mm^2^) was reported [65]. Similar release kinetics for this coating, including the initial burst release within the 8 to 48 h timeframe, were reported by Giraldo-Osorno et al. [64]. Burghardt et al. [66] also noted that the same technology released most of the Cu^2+^ ions in the first 24 h, and that a gradual decline of the Cu^2+^ release was observed until 72 h. Here, we extended the release study up to 14 days and demonstrated that Cu release was still detectable up to 14 days, albeit in small quantities. These measurements of Cu^2+^ release indicate that the amounts of Cu remaining on the surface after 72 h may be low, thereby reducing the coating’s efficiency.

### 3.8. CFU Counting by Serial Dilution Technique

**Figure 5:**
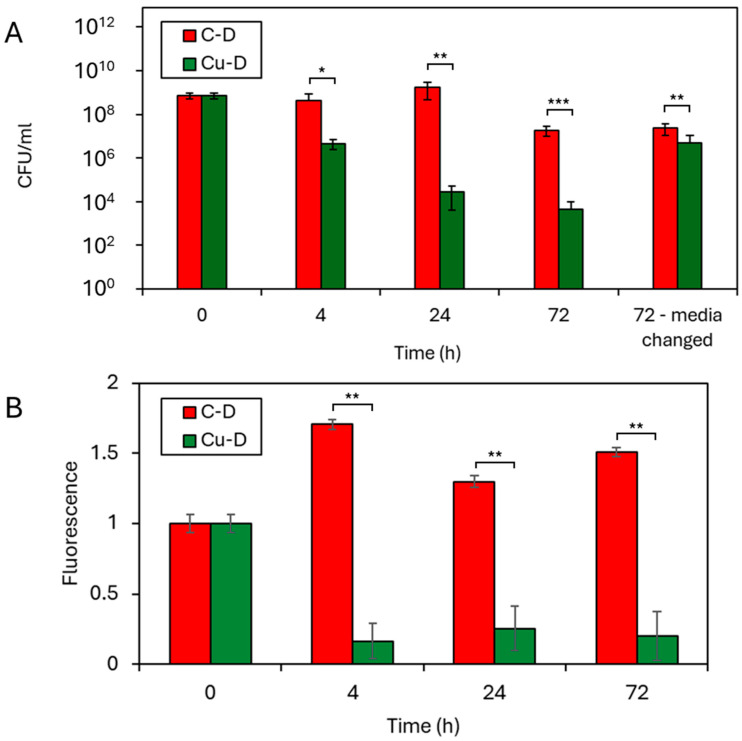
Antibacterial properties against *S. aureus* (ATCC6538) measured through (**A**) CFU count by serial dilution technique and (**B**) fluorescence (normalised by initial value) revealed by the Presto Blue analysis, for C-D and Cu-D groups. * *p*-value < 0.05; ** *p*-value < 0.01; *** *p*-value < 0.001.

The CFU counting by the serial dilution technique was used to evaluate the antibacterial activity of C-D and Cu-D samples, and the results of the analysis are shown in Figure 5A. The bacterial concentration at t = 0 h was initially estimated by OD_600_ measurement and subsequently confirmed by CFU counting, resulting in a final concentration of 7.3 × 10^8^ CFU/mL. In the negative control group (C-D samples, sterile PBS), CFU counts did not reveal any bacteria at the surface of the disc at any timepoints, confirming no cross-contamination. In contrast, CFU counts in the C-D group was 4.2 × 10^8^ CFU/mL at t = 4 h, 1.7 × 10^9^ CFU/mL at t = 24 h and 1.9 × 10^7^ CFU/mL at t = 72 h, indicating increasing bacterial colonisation on the surface of the discs during the first 24 h, followed by a slight decline at 72 h. When the media was changed, the retrieved bacterial concentration was 2.4 × 10^7^ CFU/mL at t = 72 h, showing that the change of media did not affect the population of bacteria adhering to the discs. In the Cu-D, group, the CFU counts of retrieved bacteria revealed consistent decline of the bacterial population, with 4.6 × 10^6^ CFU/mL at t = 4 h, 2.7 × 10^4^ CFU/mL at t = 24 h and 4.6 × 10^3^ CFU/mL at t = 72 h. When compared to C-D group, Cu-D discs underwent a 2 log_10_ bacterial colonisation reduction after 4 h (*p* < 0.05), a 4.8 log_10_ reduction after 24 h (*p* < 0.01), and a 3.6 log_10_ reduction after 72 h (*p* < 0.001). These reductions indicate a significant bactericidal effect in vitro, as early as 4 h, and extend to up to 24 h.

The efficacity on shorter timepoints (<4 h) are comparable to the outcomes from other studies on Cu coatings and Cu alloys against the same bacteria in vitro [69,76]. Notably, Hoene et al. [65] evaluated the same electrodeposited Cu coating from the same manufacturer, also glass-bead-blasted, and found a 3 log_10_ reduction after 4 h, but with a lower inoculation dose (5 × 10^4^ CFU/mL). Similarly, Giraldo-Osorno et al. [64], who evaluated the same coating technology revealed a higher reduction in the bacterial population after 4 h, with 3.16 and 3.96 log_10_ reduction when cultured in TSB and Roswell Park Memorial Institute 1640 (RPMI) media, but also from a lower inoculation dose (10^5^ CFU/mL). Interestingly, a study from Nie et al. [61] used a similar inoculation dose than ours (1.5 × 10^8^ CFU/mL) and observed after 3 h a 7.98 log_10_ reduction in Methicillin-resistant *S. aureus* on Cu-dotted coatings prepared via electrodeposition. This higher reduction compared to our results could be attributed to the high hydrophilicity of their coatings, resulting from the fabrication technique, which allowed bacteria to have more effective contact with Cu due to the spreading of the bacterial suspension media. Indeed, Nie et al., deposited an aluminium oxide with specific morphologies layer before depositing spherical Cu dots to increase the hydrophilicity.

**Figure 6:**
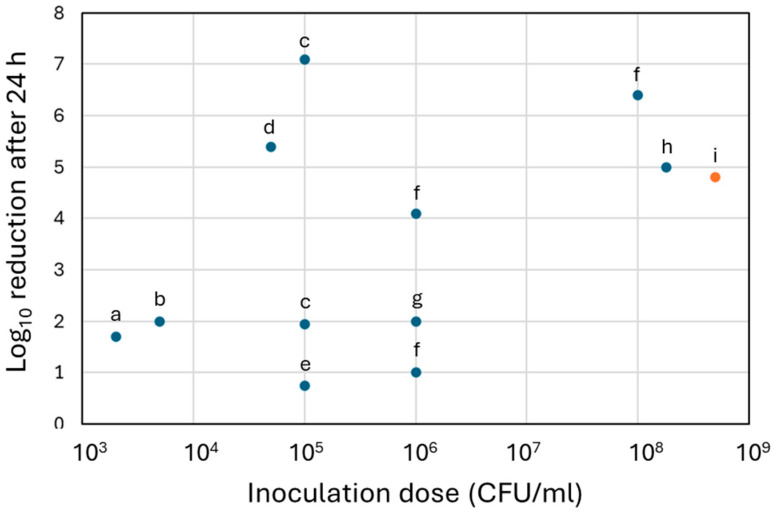
Comparison of the antibacterial activity of the Cu coating of the present study (via CFU count analysis) with other Cu coatings obtained from electrodeposition technique and reported in literature. Only the antibacterial activity after 24 h exposure to the coating was considered and plotted as function of the initial inoculum concentration. Data were used from the following references: (a) Van Hengel et al., 2020 [68], (b) Burghardt et al., 2015 [66], (c) Giraldo-Osorno et al., 2025 [64], (d) Hoene et al., 2013 [65], (e) Zhang et al., 2018 [76], (f) Ciacotich et al., 2018 [74], (g) Popova et al., 2023 [69], (h) Dawari et al., 2022 [67], (i) present study.

Regarding the results at 24 h timepoint, comparable reductions (3 to 10 log_10_) were observed in other studies on Cu coating against *S. aureus* strains [55,60], including studies with electrochemically deposited Cu coatings [65,67,74]. Figure 6 compares the reduction at 24 h observed in the present work with reductions at 24 h reported in other studies that evaluated electrodeposited Cu coatings against *S. aureus*. The inoculation dose used in the present study is the highest to have been tested on electrodeposited Cu coatings. Despite the high inoculation dose and the increased hydrophobicity of the Cu-D samples, the reduction in the bacterial growth, compared to results from literature, is promising. Our evaluation, conducted with a high inoculation dose, was complementary to studies on the same technology but using lower inoculation doses. Notably, after 24 h of exposure, Hoene et al. [65] observed 5.39 log10 reduction in *S. aureus* growth, and Giraldo-Osorno et al. [64] revealed 1.2 and 8.02 log_10_ reductions when cultured in TSB and RPMI media, respectively.

In the present work, when the bacterial media was changed every 24 h, the antibacterial effect was much less pronounced, as the bacterial population adhered to the Cu-D samples was 5.1 × 10^6^ CFU/mL, which was only 5 times less than the bacterial population in the C-D group. This can be attributed to a diminished contact killing mechanism, as the Cu is mostly released at 72 h, along with the absence of accumulated Cu in the freshly renewed medium that could have controlled the bacterial growth. Indeed, each disc released approximately 224 µg of Cu within the first 24 h according to the Cu release kinetics experiments (Figure 4), and the concentration of Cu could have exceeded the minimal bactericidal concentration (MBC) against *S. aureus* (160 µg/mL [111]). For instance, Giraldo-Osorno et al. [64] pre-conditioned two culture media (TSB and RPMI) by immersing the Cu-coated discs for 24 h before culturing the bacteria, and measured 0.78 and 9.3 log_10_ reductions after 24 h. In addition, the bacterial concentration measured at 72 h on the Cu-D samples with media changed every 24 h was close to that measured at 4 h (5.1 versus 4.6 × 10^6^ CFU/mL). The previous results on the Cu release kinetics showed that each disc released 65 µg in the first 4 h, and 19 µg between 48 h and 72 h. It is therefore likely that the remaining Cu at the surface of the disc at 72 h still contributed to the reduction in the bacterial population via the contact killing mechanism, which compensated for the diminished bactericidal effect of the lower concentration of Cu in the media.

Overall, these results indicate that the increased concentration of Cu in the medium had a bactericidal effect, in parallel to the known contact killing mechanism widely reported in literature for this type of metallic coating. Local Cu concentrations may also vary drastically around a Cu-coated device in a clinical scenario or in an in vivo context. In this in vitro assay, two extreme scenarios were tested, one with an accumulation of Cu in the media and only one bacterial challenge, and one with three bacterial challenges and a reduction of Cu ions in solutions due to the media change. The resulting observations presented here could assist the interpretations of future in vivo evaluations. Indeed, due to the combined effect of contact killing and the increasing Cu concentration around a Cu-coated device, the results obtained from animal models evaluating the efficacy of the coating may depend on the inoculation method. Bone infection models in large animals using collagen sponges to inoculate the bacteria [111,112,113,114], might not fully benefit from the contact killing mechanism. On the other hand, animal models that mimic a clinical scenario by inoculating the bacteria directly on or around the bactericidal implant can demonstrate the combined effect of contact killing and increasing Cu concentration around the device.

### 3.9. Presto Blue Assay

The metabolic activity of bacteria cultured on C-D and Cu-D was evaluated at t = 4 h, 24 h, and 72 h after incubation using the Presto Blue assay (Figure 5B). When compared to C-D samples, the fluorescence of Cu-D samples exhibited a 6-fold drop at 4 h, a 5-fold decrease at 24 h, and a 7-fold decrease at 72 h, with a statistical significance level below 0.01 at all timepoints, indicating a significant reduction in bacterial metabolic activity for the Cu-D group when compared to the C-D group. The reduction in both the metabolic activity and bacterial concentration supports the antibacterial efficacy of the coating.

### 3.10. SEM Imaging of S. aureus Colonisation

**Figure 7:**
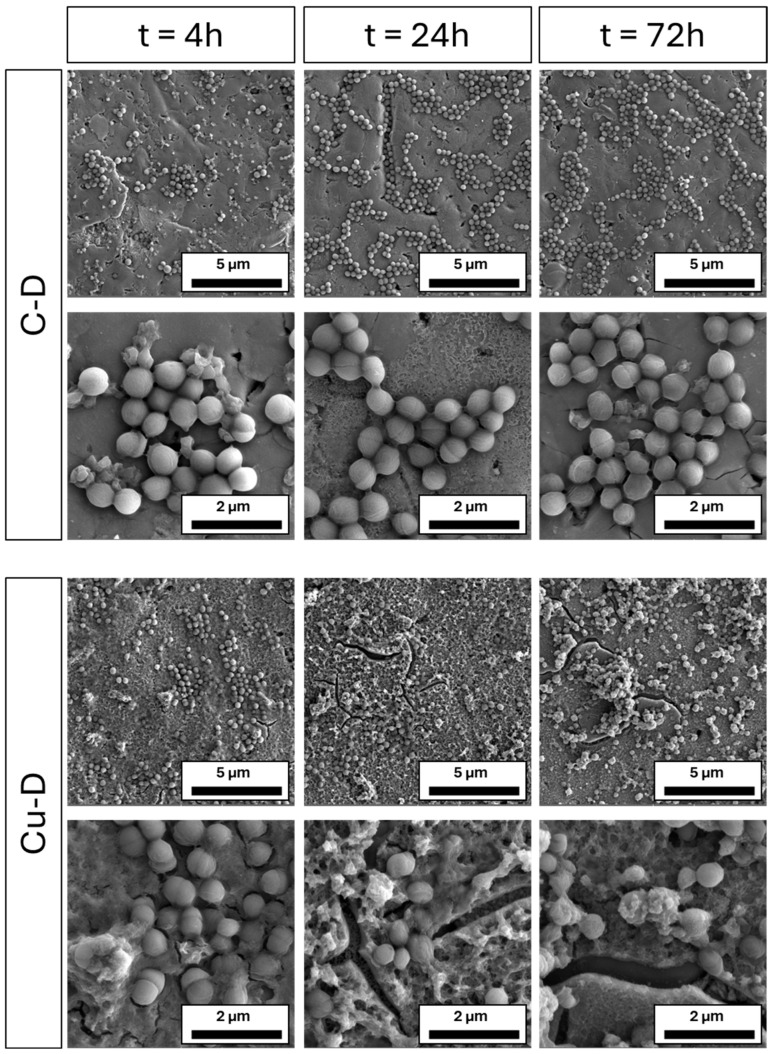
Representative SEM images at various time intervals, at 20,000 and 50,000 magnifications, demonstrating the alterations in the morphology of *S. aureus* on Cu-D samples.

*S. aureus* colonisation on C-D and Cu-D surfaces throughout different time intervals (4 h, 24 h, and 72 h) was observed with SEM imaging (Figure 7). At 4 h, both C-D and Cu-D samples exhibited a growing bacterial population at the different locations of the samples. After 24 h and 72 h, bacterial growth in C-D samples could be observed, with an increased population distributed in clusters, compared to the 4 h timepoint where bacteria were sparsely distributed. At each timepoint, there was no change in the morphology of *S. aureus* for this group. On the Cu-D samples, some bacteria were also observed on the surface for all timepoints, but no clustering of bacteria was observed at 24 h and after. At 24 h, the fragmented cytoskeletons of the bacteria suggested the appearance of bacterial damage or stress. After 72 h, the bacteria on the Cu-D samples were scarcer and more irregularly shaped. The observed changes in bacterial morphology can be attributed to the presence of Cu on the surface, which damages the bacterial membranes. These observations are consistent with the outcomes from the CFU counting and Presto Blue analysis, where antibacterial efficacy was demonstrated.

### 3.11. Live/Dead Imaging

**Figure 8:**
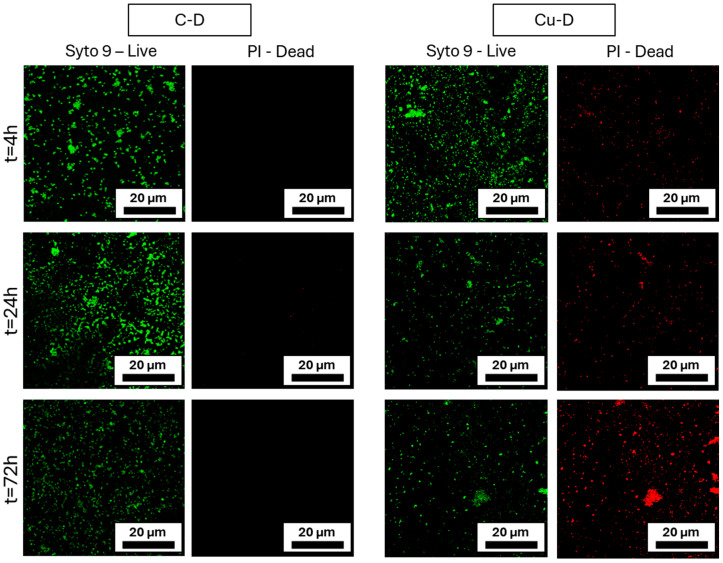
Live-dead confocal microscopy images captured at different timepoints, revealing the distribution of live and dead *S. aureus* on C-D and Cu-D samples.

At each timepoint, C-D and Cu-D surfaces were stained with Syto9 and PI to differentiate viable and dead bacteria, respectively, and then imaged using a confocal microscope (Figure 8). In the C-D samples group, Syto 9 staining in images taken at 4 h revealed the presence of viable bacteria, and those taken at 24 h showed a growing population. At 24 h and 72 h, a vibrant green light was observed, attributed to bacterial growth and a higher cell density. The absence of red colour indicated that no evidence of bacterial death was detected with PI staining in this group. The Cu-D samples exhibited a similar concentration of viable bacteria after 4 h, along with a minimal presence of dead bacteria. At 24 h, a rise in the number of dead bacteria and a decrease in the number of live bacteria were observed. At 72 h, the trend was similar, with an increase in the presence of vibrant red fluorescence regions. These observations demonstrated that the Cu-D group exhibited antibacterial properties in comparison to the C-D group. The live/dead results combined with the SEM observations from the previous section indicated that the bacteria were able to adhere to the surface despite its hydrophobicity, and that the number of killed bacteria was rising. These observations confirmed the previous analyses obtained in the CFU count, Presto Blue, and SEM analysis.

In summary, compared to recent studies on the same electrodeposited Cu coating [64,65], this work extended surface characterisation to elemental analysis using XPS and SEM-EDS, and bactericidal evaluation by testing a higher bacterial concentration and incorporating an additional timepoint. Elemental analysis confirmed the presence of Cu on the Cu-D surface and provided quantitative measurements. Investigations into surface roughness and contact angle revealed modifications to wetting and topological properties that could affect bacterial adhesion and proliferation. Particularly, Cu-D samples exhibited enhanced hydrophobicity. The release profile of Cu ions from Cu-D samples showed a burst release in the first few days, followed by a progressive decrease up to 14 days. This release profile indicates that the efficiency of the coating probably decreases after three days, which can help make biomedical devices using this technology efficient in preventing SSIs by reducing the bacterial population in the days that follow the surgery. Additionally, the antibacterial properties of Cu-D surfaces were evaluated in vitro against a high dose of *S. aureus.* The results of antibacterial activity against *S. aureus* showed significant 4.8 and 3.6 log_10_ reductions at 24 h and 72 h, which rank among the highest bacterial reduction rates reported in the literature, despite the high inoculum selected in this study. Significant bacterial colonisation reductions were also observed in the Presto Blue assay, and two imaging techniques (SEM and live-dead imaging with confocal) showed the presence of dead bacteria with damaged outer membranes, thereby confirming the quantitative results obtained with CFU count analysis.

The antibacterial properties demonstrated in this work, particularly with a high inoculum, open the door for future pre-clinical investigations in the orthopaedic field. As mentioned earlier, fracture-related SSIs are a major problem in the treatment of tibial open fractures. Currently, the golden standard treatment for tibial open fractures is Ti nail implantation (or intramedullary nailing) [112]. 73% of infections in open fractures occur pre- and intra-operatively by bacteria penetrating the trauma itself and can be initiated by inoculums as little as 10^3^ CFU [8,113]. The use of Cu-coated nailing systems could drastically reduce the prevalence of SSIs by eradicating bacteria upon burst release of Cu^2+^ ions in the 72 h following the surgical procedure. The performances of the technology in in vivo studies must however be further evaluated. For instance, Hoene et al. [65] measured the cytotoxic effect of the technology in a rat model but using Cu concentration in the coatings much lower than those reported in the present study, and local tissue reactions are still unknown for higher Cu concentration. Cytotoxic assessment with higher Cu concentration is essential since in vitro investigations showed that Cu coatings releasing 0.5 mM (317 µm/mL) of Cu could be cytotoxic for mesenchymal stem cells [66]. The most complete in vivo evaluation of the Cu coating technology to date is a work from Prinz et al. [114] that investigated the osteo-regenerative and antibacterial properties of intramedullary nails in a rabbit bone tibial fracture model. Using a 100 µL inoculum of *S. aureus* concentrated at 10^5^ CFU/mL, they could show that the Cu-coated nails successfully eradicated the bacteria, and no bacteria were retrieved after 28 days. In comparison, control animals developed infections where up to 10^9^ CFU/mL were retrieved from the surface of the implants. In parallel, radiographic and histological examinations revealed increased callus formation in animals with a Cu-coated nail, regardless of the bacterial inoculation. The concentration of Cu used in this study was therefore high enough to combine two important biological functions in open fracture treatments, e.g., to combat bacteria and to stimulate bone healing. The Cu concentration measured in the blood of the animals were also ten times smaller than those found cytotoxic in vitro (317 µm/mL). However, evaluation in large animal models remains essential to better estimate local tissue reactions and bacteria reduction in clinical scenario.

One challenge is to develop an appropriate osteomyelitis model that replicates the contamination scenario in a clinical context and is adapted to the killing mechanism of Cu. For instance, most tibial osteomyelitis models are used to test the efficacy of antibiotics-coated nails, for which the antibiotics is released over weeks. Such models rely on the long-term diffusion of the antibiotics, and inoculation is conducted via insertion of a contaminated collagen sponge [24,115,116]. Such an inoculation technique could limit the contact killing mechanism observed with Cu coatings or isolate the bacteria from the 72 h-burst release during the degradation of the sponge, thereby underestimating the efficacy of the coating. To address these concerns, our research team initiated the development of a new large animal osteomyelitis model. Further investigations of the Cu coating will then be conducted via an in vivo assessment in the new osteomyelitis model on sheep. Sheep have an osseous microarchitecture, bone composition, remodelling capacity, and morphological and biomechanical properties similar to those of human [117]. These similarities allow testing human size intramedullary nails, which will unveil new insights on the in vivo Cu release rate and antibacterial properties of the Cu-coating technology.

## 4. Conclusions

The integration of Cu into TiO_2_ coatings on Ti substrates represents a paradigm shift in biomedical surface engineering, particularly in the ongoing battle against implant-associated infections. The in vitro antimicrobial efficacy of such TiO_2_-Cu coated Ti surfaces is not merely a functional enhancement; it is a biomaterial innovation that aligns biocompatibility with potent, localised antimicrobial activity.

This study characterised the physicochemical properties of a new bactericidal surface consisting of a TiO_2_-Cu coating deposited on Ti alloy by a novel technology. The study also evaluated the antibacterial properties of the Cu-coated surface through a series of assays. Overall, these results demonstrated the in vitro bactericidal properties of the technology and justified the need for further development in animal model studies.

In addition to future in vivo investigation, which will be a critical step in assessing the antibacterial effect of the coating in a biologically relevant environment and evaluating its potential for clinical applications, further in vitro investigations could expand the scope of this study. A Cu coating exhibiting the same hydrophilicity as the control group could reveal the role of the hydrophilicity on the bactericidal efficacy. A limitation of the study is the duration of the bacterial reduction analysis. Here, the assays were conducted up to 72 h, but a further evaluation, with timepoints up to one week, could determine if re-population of *S. aureus* on the surface, possibly free of Cu, is possible. If no regrowth is observed, this would indicate potent and long-lasting antibacterial effects. Finally, practical and fundamental questions have arisen during this work. They will inspire future studies on this type of coating, such as its efficacy against other bacterial strains, or whether the release kinetics or bactericidal efficacy is affected by the volume of Cu deposited on the surface.

The TiO_2_-Cu-coated Ti surface stands at the convergence of materials science, microbiology, and clinical innovation. The development of Cu coatings with intrinsic antibacterial properties for biomedical implant applications has the potential to impact the healthcare system and society at large. As in vitro results continue to suggest its efficacy, the challenge now lies in refining its interface with host biology and manufacturing ecosystems. Upon application of this technology to Ti biomedical implants, commercialization of the product, and successful translation into clinical practices, the management of infections in orthopaedic surgery and regenerative medicine could be significantly improved. Future developments could pave the way towards bactericidal implants and improved healthcare delivery. It must be ensured that the leap from bench to bedside is not only scientifically sound but also surgically transformative.

## Figures and Tables

**Table 1:** Bactericidal potential of metallic coatings prepared through electrodeposition processes. BHI: Brain heart infusion, MRSA: Methicillin-resistant *S. aureus*, PBS: Phosphate-buffered saline solution.

Coating	Deposition Process	Substrate Characteristics	In Vitro Model Design	Outcomes	Reference
Cu	Plasma electrolytic oxidation	Ti6Al4V Plates, 5 × 5 × 1 mm	*S. aureus* CFU count, 2, 4, 6, 8 and 24 h timepoints 5 × 10^4^ CFU/mL dose	Adhered bacteria on samples decreased to undetectable levels after 2 h and further timepoints. Compared to control samples, this corresponded to 3 log_10_, 4 log_10_, 5.39 log_10_ reductions after 4 h, 6 h and 24 h.	Hoene et al., 2013 [65]
	Plasma electrolytic oxidation	Ti6Al4V Plates, 5 × 5 × 1 mm Cu concentrations 0.05–0.5 mM	*S. aureus* CFU count, 6, 24 and 48 h timepoints 5 × 10^3^ CFU/mL dose	Reduction in bacterial growth was more significant with higher Cu concentration. For 0.5 mM, 2 log_10_ reductions were observed at 24 and 48 h.	Burghardt et al., 2015 [66]
	Plasma electrolytic oxidation	Ti6Al4V Discs, ⌀ 12 mm, height 3 mm	*S. aureus* CFU count, 4 and 24 h timepoints 10^5^ CFU/mL dose	No bactericidal effect observed after 4 h. 1.95 log_10_ (98.86%) to 7.10 log_10_ (99.99%) reductions after 24 h, depending on culture media.	Giraldo-Osorno et al., 2025 [64]
	Electroplating	Tungsten carbide-cobalt plates	*S. aureus* CFU count, 24 h timepoint 1.8 × 10^8^ CFU/mL dose	5.0 log_10_ reduction.	Dawari et al., 2022 [67]
	Plasma electrolytic oxidation	Ti6Al4V Rods, ⌀ 0.5 mm, height 10 mm	*S. aureus* CFU count, overnight 2 × 10^3^ CFU/mL dose	1.7 log_10_ reduction.	Van Hengel et al., 2020 [68]
	Plasma electrolytic oxidation	Grade 4 Ti Plates, 15 × 15 × 3 mm	MRSA and *E. coli* CFU count, 6 and 24 h timepoints 10^5^–10^6^ CFU/ml	6 log_10_ reduction against *E. coli* after 6 h. 1 log_10_ (90%) and 2 log_10_ (99%) reductions against MRSA after 6 and 24 h.	Popova et al., 2023 [69]
	Plasma electrolytic oxidation	AA1100 Al Plates, 25 × 25 × 5 mm	MRSA, *E. coli* and *E. faecium* CFU count, 1, 2 and 3 h timepoint. 1.5 × 10^8^ CFU/mL dose	For all strains, bacterial concentration decreased faster than control Al plates. At 3 h, 1.70 log_10_, 7.98 log_10_, and 1.86 log_10_ reductions were observed for *E. coli*, MRSA and *E. faecium*.	Nie et al., 2010 [61]
	Electrophoresis	304 stainless steel Plates, 20 × 20 × 1 mm	*S. aureus* and *E. coli* Time-of-contact, 5, 10, 15, 20, 25 and 30 min 10^5^ CFU dose	Complete reduction in *E. coli* after 5 min of exposure, and of *S. aureus* after 10 min (5.0 log_10_ reduction).	Isa et al., 2017 [62]
	Electroplating	Al Plates, 100 mm^2^	*E. coli* CFU count, 6 h timepoint 10^7^ CFU/mL dose	1.4 log_10_ (96%) reduction.	Augustin et al., 2016 [70]
	Ultrasonic-assisted electrophoresis	Al_2_O_3_-coated Al	*E. coli* CFU count, 1 h timepoint 10^5^ CFU/mL dose	5.0 log_10_ reduction.	Chen et al., 2024 [71]
	Electroplating	Carbonised polyaramid Plates, 10 × 10 mm	*E. coli* and *B. subtilis* CFU count, 75 min timepoint	3.0 log_10_ reduction for both strains.	Mamleyev et al., 2020 [72]
	Electrophoresis	Microstructured copper foils, 2.5 × 2.5 × 0.05 cm	*E. coli.* Time-of-contact, 20, 40, 60, 100 and 180 min 2 × 10^8^ CFU/mL dose	Complete reduction in *E. coli* after 60 min of exposure, or 100 min for surface non-microstructured (8.3 log_10_ reduction).	Zeiger et al., 2014 [73]
Cu-Ag	Plasma electrolytic oxidation	Ti6Al4V Rods, ⌀ 0.5 mm, height 10 mm	*S. aureus* CFU count, overnight 2 × 10^3^ CFU/mL dose	8.4 log_10_ reduction.	Van Hengel et al., 2020 [68]
	Electroplating	AISI 316L steel Plates, 10 × 20 × 1 mm	*S. aureus* and *E. coli* CFU count, 0.5, 4 and 24 h timepoints 10^6^ and 10^8^ CFU/mL doses in PBS and BHI broth	For *S. aureus*, gradual bacterial growth reductions were observed across timepoints. At 24 h, 4.1 log_10_ reduction for small dose and 6.4 log_10_ reduction for high dose in PBS were observed, but only 1.0 log_10_ reduction (90.0%) for small dose in broth. For *E. coli*, 2.5–2.7 log_10_ reductions were observed for all timepoints for small dose in PBS.	Ciacotich et al., 2018 [74]
Cu, Cu-SiC	Pulse reverse electrodeposition	Annealed Cu Plates, 2 cm × 2 cm × 100 μm	*E. coli* and *B. subtilis* CFU count, 4, 8, 12, 16, 20 and 24 h timepoints 1.5 × 10^8^ CFU dose	Only 1.11 log_10_ reductions for both *E. coli* and *B. subtilis* at 24 h with the Cu coating, but 7.14 and 10 log_10_ reductions at 24 h with the Cu-SiC coating.	Banthia et al., 2019 [75]
Cu, Cu-Zn	Plasma electrolytic oxidation	Ti6Al4V Discs, ⌀ 14 mm, height 2 mm	*S. aureus* CFU count, 6 and 24 h timepoints 10^5^ CFU/mL dose	78% and 82% growth reduction at 6 and 24 h for the Cu coating. Up to 90% and 92% reduction at 6 and 24 h for the Cu coating with the highest concentration of Zn.	Zhang et al., 2018 [76]
Cu-Sn and Cu-Sn-TiO_2_	Ultrasonic-assisted electrophoresis	Stainless steel Plates, 4 cm^2^ Different concentrations of Cu, Sn and TiO_2_	*E. coli* CFU count, 20 and 30 min timepoints 9.2 × 10^5^ CFU dose	No reduction in Cu-Sn coatings. Highest reduction after 30 min was 57%, for 82.4% Cu−10% Sn−7.6% Ti. Highest reductions rates observed with combination with UV exposure.	Kharitonov et al., 2021 [77]
Ag	Electroplating	304 stainless steel Plates, 50 × 50 × 1 mm	*S. aureus* and *E. coli* CFU count, 72 h timepoint 6 × 10^5^ CFU/mL dose	Antibacterial rate against *E.coli.* and *S. aureus.* reached 99.88% (2.9 log_10_) and 97.92% (1.7 log_10_), respectively.	Wang et al., 2022 [78]
	Electrophoresis	Stainless steel Plates, 3.5 × 2.5 cm	*S. aureus* and *E. coli* Zone of inhibition	Inhibition zone diameters were 1.5–2 cm *against S. aureus* and 1.5–1.9 cm against *E. coli* (depending on the deposition parameters investigated in the study).	El Sayed et al., 2022 [79]
	Plasma electrolytic oxidation	Ti6Al4V Rods, ⌀ 0.5 mm, height 10 mm	*S. aureus* CFU count, overnight 2 × 10^3^ CFU/mL dose	8.4 log_10_ reduction.	Van Hengel et al., 2020 [68]
	Electrophoresis	Ti6Al4V Discs, ⌀ 6 mm, height 1 mm	*P. gingivalis* and *P. intermedia* CFU count, 24 h timepoint 5 and 30 nm particle size 100, 200 and 300 ppm particle concentration.	Decrease in bacterial population increased gradually with the concentration of Ag particles. Higher reduction was observed with smaller particles. Maximum reductions were 29.9% and 26%, for the 5 nm/300 ppm and 30 nm/300 ppm groups.	Kirmanidou et al., 2019 [80]
Zn-Ag	Electroplating	AISI 1018 steel Plates, 10 × 15 cm	*S. aureus* and *E. coli* Time-of-contact, 1, 15 and 30 min 0–14.0 mg/cm^3^ Ag concentrations	Zn with 4.3 mg/cm^3^ Ag reduced *E. coli* and *S. aureus* growth by 94.1% and 91.0% after 1 min, and 95.4% and 98.4%, after 30 min. Results were similar for higher concentrations of Ag.	Reyes-Vidal et al., 2015 [81]
Zn	Electroplating	Tungsten carbide-cobalt plates	*S. aureus* CFU count, 24 h timepoint 1.8 × 10^8^ CFU/mL dose	2.0 log_10_ reduction.	Dawari et al., 2022 [67]
	Electroplating	AISI 1018 steel Plates, 10 × 15 cm	*S. aureus* and *E. coli* Time-of-contact, 1, 15 and 30 min	Zn alone reduced *E. coli* and *S. aureus* growth by 87.0% and 89.5% after 1 min, and 80.5% and 83.73%, after 30 min.	Reyes-Vidal et al., 2015 [81]
Zn, Sr, Zn-Sr	Microarc oxidation	TA2 Ti Discs, ⌀ 14.75 mm, height 1 mm	*S. aureus* CFU count, 24 h timepoint 10^4^ CFU/mL dose	60% and 80% reduction in bacterial growth on Sr and Zn-Sr coatings. Complete reduction of bacterial growth on Zn coatings (4.0 log_10_ reduction).	Zhao et al., 2019 [82]
ZnO	Electrophoresis	SAE 1020 carbon steel Bar, ⌀ 12 mm, height 50 mm	MRSA and *E. coli.* Zone of inhibition 20, 30, 40 and 50 ppm particle concentration	Bacterial reductions ranged from 71.4% (20 ppm particle concentration) to 99.97% (50 ppm particle concentration).	Uribe et al., 2020 [83]
MgO	Electrophoresis	Mg Plates, 10 × 10 mm	*S. aureus* CFU count, 24 h timepoint 6 × 10^6^ CFU/mL dose	6.9 log_10_ reduction compared to Ti control substrate, and 3.9 log_10_ reduction compared to Mg control substrate.	Lin et al., 2020 [52]

**Table 2:** Groups and timepoints evaluated in the antibacterial assay.

Group		Timepoints & Media Change
Negative control	C-D sample No bacteria	4 h, 24 h, 72 h No media change
		72 h Media change at 24 h and 48 h
Positive control	C-D sample *S. aureus*, 1–5 × 10^8^ CFU/mL	4 h, 24 h, 72 h No media change
		72 h Media change at 24 h and 48 h
Experimental	Cu-D sample *S. aureus*, 1–5 × 10^8^ CFU/mL	4 h, 24 h, 72 h No media change
		72 h Media change at 24 h and 48 h

## Data Availability

The raw data supporting the conclusions of this article will be made available by the authors on request.

## Supplementary Materials

The following supporting information can be downloaded at: https://www.mdpi.com/article/10.3390/nano15221742/s1.

## References

[1] Meroni G., Tsikopoulos A., Tsikopoulos K., Allemanno F., Martino P.A., Soares Filipe J.F. (2022). A journey into animal models of human osteomyelitis: A review. Microorganisms.

[2] Miclau T. (2020). Open fracture management: Critical issues. OTA Int..

[3] Hoekstra H., Smeets B., Metsemakers W.J., Spitz A.C., Nijs S. (2017). Economics of open tibial fractures: The pivotal role of length-of-stay and infection. Health Econ. Rev..

[4] Castillo I.A., Heiner J.A., Meremikwu R.I., Kellam J., Warner S.J. (2023). Where are we in 2022? A summary of 11,000 open tibia fractures over 4 decades. J. Orthop. Trauma.

[5] Li J., Wang Q., Lu Y., Feng Q., He X., Li Z., Zhang K. (2020). Relationship between time to surgical debridement and the incidence of infection in patients with open tibial fractures. Orthop. Surg..

[6] Schwarz E.M., Parvizi J., Gehrke T., Aiyer A., Battenberg A., Brown S.A., Callaghan J.J., Citak M., Egol K., Garrigues G.E. (2019). 2018 international consensus meeting on musculoskeletal infection: Research priorities from the general assembly questions. J. Orthop. Res..

[7] Bachoura A., Guitton T.G., Smith R.M., Vrahas M.S., Zurakowski D., Ring D. (2011). Infirmity and injury complexity are risk factors for surgical-site infection after operative fracture care. Clin. Orthop. Relat. Res..

[8] Trampuz A., Zimmerli W. (2006). Diagnosis and treatment of infections associated with fracture-fixation devices. Injury.

[9] Dicks K.V., Lewis S.S., Durkin M.J., Baker A.W., Moehring R.W., Chen L.F., Sexton D.J., Anderson D.J. (2014). Surveying the surveillance: Surgical site infections excluded by the January 2013 updated surveillance definitions. Infect. Control Hosp. Epidemiol..

[10] Thakore R.V., Greenberg S.E., Shi H., Foxx A.M., Francois E.L., Prablek M.A., Nwosu S.K., Archer K.R., Ehrenfeld J.M., Obremskey W.T. (2015). Surgical site infection in orthopedic trauma: A case-control study evaluating risk factors and cost. J. Clin. Orthop. Trauma.

[11] Kurtz S.M., Lau E., Watson H., Schmier J.K., Parvizi J. (2012). Economic burden of periprosthetic joint infection in the United States. J. Arthroplast..

[12] Metsemakers W.J., Handojo K., Reynders P., Sermon A., Vanderschot P., Nijs S. (2015). Individual risk factors for deep infection and compromised fracture healing after intramedullary nailing of tibial shaft fractures: A single centre experience of 480 patients. Injury.

[13] Galvain T., Chitnis A., Paparouni K., Tong C., Holy C.E., Giannoudis P.V. (2020). The economic burden of infections following intramedullary nailing for a tibial shaft fracture in England. BMJ Open.

[14] Jernigan J.A. (2004). Is the burden of *Staphylococcus aureus* among patients with surgical-site infections growing?. Infect. Control Hosp. Epidemiol..

[15] Anderson D.J., Sexton D.J., Kanafani Z.A., Auten G., Kaye K.S. (2007). Severe surgical site infection in community hospitals: Epidemiology, key procedures, and the changing prevalence of methicillin-resistant *Staphylococcus aureus*. Infect. Control Hosp. Epidemiol..

[16] McConoughey S.J., Howlin R., Granger J.F., Manring M.M., Calhoun J.H., Shirtliff M., Kathju S., Stoodley P. (2014). Biofilms in periprosthetic orthopedic infections. Future Microbiol.

[17] Arciola C.R., Campoccia D., Ehrlich G.D., Montanaro L. (2015). Biofilm-based implant infections in orthopaedics. Adv. Exp. Med. Biol..

[18] Zimmerli W., Sendi P. (2017). Orthopaedic biofilm infections. APMIS J. Pathol. Microbiol. Immunol..

[19] Llor C., Bjerrum L. (2014). Antimicrobial resistance: Risk associated with antibiotic overuse and initiatives to reduce the problem. Ther. Adv. Drug Saf..

[20] Raschke M.J., Rosslenbroich S.B., Fuchs T.F., Rommens P.M., Hessmann M.H. (2015). Antibiotic coated nails. Intramedullary Nailing: A Comprehensive Guide.

[21] Goodman S.B., Yao Z., Keeney M., Yang F. (2013). The future of biologic coatings for orthopaedic implants. Biomaterials.

[22] Bohara S., Suthakorn J. (2022). Surface coating of orthopedic implant to enhance the osseointegration and reduction of bacterial colonization: A review. Biomater. Res..

[23] Boot W., Foster A.L., Guillaume O., Eglin D., Schmid T., D’Este M., Zeiter S., Richards R.G., Moriarty T.F. (2022). An Antibiotic-Loaded Hydrogel Demonstrates Efficacy as Prophylaxis and Treatment in a Large Animal Model of Orthopaedic Device-Related Infection. Front. Cell. Infect. Microbiol..

[24] Foster A.L., Boot W., Stenger V., D’Este M., Jaiprakash A., Eglin D., Zeiter S., Richards R.G., Moriarty T.F. (2021). Single-stage revision of MRSA orthopedic device-related infection in sheep with an antibiotic-loaded hydrogel. J. Orthop. Res..

[25] Stewart S., Barr S., Engiles J., Hickok N.J., Shapiro I.M., Richardson D.W., Parvizi J., Schaer T.P. (2012). Vancomycin-modified implant surface inhibits biofilm formation and supports bone-healing in an infected osteotomy model in sheep: A proof-of-concept study. J. Bone Jt. Surg. Am..

[26] Gimeno M., Pinczowski P., Mendoza G., Asín J., Vázquez F.J., Vispe E., García-Álvarez F., Pérez M., Santamaría J., Arruebo M. (2018). Antibiotic-eluting orthopedic device to prevent early implant associated infections: Efficacy, biocompatibility and biodistribution studies in an ovine model. J. Biomed. Mater. Res. Part B Appl. Biomater..

[27] Jorge-Mora A., Amhaz-Escanlar S., Fernandez-Pose S., García-Iglesias A., Mandia-Mancebo F., Franco-Trepat E., Guillán-Fresco M., Pino-Minguez J. (2019). Commercially available antibiotic-laden PMMA-covered locking nails for the treatment of fracture-related infections—A retrospective case analysis of 10 cases. J. Bone Jt. Infect..

[28] Inoue D., Kabata T., Ohtani K., Kajino Y., Shirai T., Tsuchiya H. (2017). Inhibition of biofilm formation on iodine-supported titanium implants. Int. Orthop..

[29] Huang Y.Y., Choi H., Kushida Y., Bhayana B., Wang Y., Hamblin M.R. (2016). Broad-spectrum antimicrobial effects of photocatalysis using titanium dioxide nanoparticles are strongly potentiated by addition of potassium iodide. Antimicrob. Agents Chemother..

[30] Bosch E.H., van Doorne H., de Vries S. (2000). The lactoperoxidase system: The influence of iodide and the chemical and antimicrobial stability over the period of about 18 months. J. Appl. Microbiol..

[31] Zhao Z. (2022). Nanosurface modification of Ti64 implant by anodic fluorine-doped alumina/titania for orthopedic application. Mater. Chem. Phys..

[32] Yamaguchi S., Le P.T.M., Shintani S.A., Takadama H., Ito M., Ferraris S., Spriano S. (2021). Iodine-loaded calcium titanate for bone repair with sustainable antibacterial activity prepared by solution and heat treatment. Nanomaterials.

[33] Inoue D., Kabata T., Kajino Y., Shirai T., Tsuchiya H. (2019). Iodine-supported titanium implants have good antimicrobial attachment effects. J. Orthop. Sci..

[34] Daeschlein G. (2013). Antimicrobial and antiseptic strategies in wound management. Int. Wound J..

[35] Lollobrigida M., Filardo S., Sessa R., Di Pietro M., Bozzuto G., Molinari A., Lamazza L., Vozza I., De Biase A. (2019). Antibacterial activity and impact of different antiseptics on biofilm-contaminated implant surfaces. Appl. Sci..

[36] Tripathy A., Sen P., Su B., Briscoe W.H. (2017). Natural and bioinspired nanostructured bactericidal surfaces. Adv. Colloid Interface Sci..

[37] Oopath S.V., Baji A., Abtahi M., Luu T.Q., Vasilev K., Truong V.K. (2023). Nature-inspired biomimetic surfaces for controlling bacterial attachment and biofilm development. Adv. Mater. Interfaces.

[38] Beddoes C.M., Case C.P., Briscoe W.H. (2015). Understanding nanoparticle cellular entry: A physicochemical perspective. Adv. Colloid Interface Sci..

[39] Wang L., Guo X., Zhang H., Liu Y., Wang Y., Liu K., Liang H., Ming W. (2022). Recent advances in superhydrophobic and antibacterial coatings for biomedical materials. Coatings.

[40] Linklater D.P., Ivanova E.P. (2022). Nanostructured antibacterial surfaces—What can be achieved?. Nano Today.

[41] Lemire J.A., Harrison J.J., Turner R.J. (2013). Antimicrobial activity of metals: Mechanisms, molecular targets and applications. Nat. Rev. Microbiol..

[42] Turner R.J. (2017). Metal-based antimicrobial strategies. Microb. Biotechnol..

[43] Birkett M., Dover L., Cherian Lukose C., Wasy Zia A., Tambuwala M.M., Serrano-Aroca Á. (2022). Recent advances in metal-based antimicrobial coatings for high-touch surfaces. Int. J. Mol. Sci..

[44] Zhang E., Zhao X., Hu J., Wang R., Fu S., Qin G. (2021). Antibacterial metals and alloys for potential biomedical implants. Bioact. Mater..

[45] Fordham W.R., Redmond S., Westerland A., Cortes E.G., Walker C., Gallagher C., Medina C.J., Waecther F., Lunk C., Ostrum R.F. (2014). Silver as a bactericidal coating for biomedical implants. Surf. Coat. Technol..

[46] Santo C.E., Quaranta D., Grass G. (2012). Antimicrobial metallic copper surfaces kill StapShylococcus haemolyticus via membrane damage. Microbiologyopen.

[47] Gudkov S.V., Burmistrov D.E., Serov D.A., Rebezov M.B., Semenova A.A., Lisitsyn A.B. (2021). A mini review of antibacterial properties of ZnO nanoparticles. Front. Phys..

[48] Read S.A., Obeid S., Ahlenstiel C., Ahlenstiel G. (2019). The role of zinc in antiviral immunity. Adv. Nutr..

[49] Mijnendonckx K., Leys N., Mahillon J., Silver S., Van Houdt R. (2013). Antimicrobial silver: Uses, toxicity and potential for resistance. Biometals.

[50] Liu Q., Li A., Liu S., Fu Q., Xu Y., Dai J., Li P., Xu S. (2023). Cytotoxicity of biodegradable zinc and its alloys: A systematic review. J. Funct. Biomater..

[51] Kumar K., Gill R., Batra U. (2018). Challenges and opportunities for biodegradable magnesium alloy implants. Mater. Technol..

[52] Lin J., Nguyen N.-Y.T., Zhang C., Ha A., Liu H.H. (2020). Antimicrobial properties of MgO nanostructures on magnesium substrates. ACS Omega.

[53] Vincent M., Hartemann P., Engels-Deutsch M. (2016). Antimicrobial applications of copper. Int. J. Hyg. Environ. Health.

[54] Vincent M., Duval R.E., Hartemann P., Engels-Deutsch M. (2018). Contact killing and antimicrobial properties of copper. J. Appl. Microbiol..

[55] Grass G., Rensing C., Solioz M. (2011). Metallic copper as an antimicrobial surface. Appl. Environ. Microbiol..

[56] Butler J.P. (2018). Effect of copper-impregnated composite bed linens and patient gowns on healthcare-associated infection rates in six hospitals. J. Hosp. Infect..

[57] Noyce J.O., Michels H., Keevil C.W. (2006). Potential use of copper surfaces to reduce survival of epidemic meticillin-resistant *Staphylococcus aureus* in the healthcare environment. J. Hosp. Infect..

[58] Bryce E.A., Velapatino B., Akbari Khorami H., Donnelly-Pierce T., Wong T., Dixon R., Asselin E. (2020). In vitro evaluation of antimicrobial efficacy and durability of three copper surfaces used in healthcare. Biointerphases.

[59] Charles M.K., Williams T.C., Nakhaie D., Woznow T., Velapatino B., Lorenzo-Leal A.C., Bach H., Bryce E.A., Asselin E. (2023). In vitro assessment of antibacterial and antiviral activity of three copper products after 200 rounds of simulated use. BioMetals.

[60] Montero D.A., Arellano C., Pardo M., Vera R., Gálvez R., Cifuentes M., Berasain M.A., Gómez M., Ramírez C., Vidal R.M. (2019). Antimicrobial properties of a novel copper-based composite coating with potential for use in healthcare facilities. Antimicrob. Resist. Infect. Control.

[61] Nie Y., Kalapos C., Nie X., Murphy M., Hussein R., Zhang J. (2010). Superhydrophilicity and antibacterial property of a Cu-dotted oxide coating surface. Ann. Clin. Microbiol. Antimicrob..

[62] Isa N.N.C., Mohd Y., Mohamad S.A.S., Zaki M.H.M. (2017). Antibacterial activity of copper coating electrodeposited on 304 stainless steel substrate. AIP Conference Proceedings.

[63] Bharadishettar N., Bhat K.U., Bhat Panemangalore D. (2021). Coating technologies for copper based antimicrobial active surfaces: A perspective review. Metals.

[64] Giraldo-Osorno P.M., Turner A.B., Barros S.M., Büscher R., Guttau S., Asa’ad F., Trobos M., Palmquist A. (2025). Anodized Ti6Al4V-ELI, electroplated with copper is bactericidal against *Staphylococcus aureus* and enhances macrophage phagocytosis. J. Mater. Sci. Mater. Med..

[65] Hoene A., Prinz C., Walschus U., Lucke S., Patrzyk M., Wilhelm L., Neumann H.G., Schlosser M. (2013). In vivo evaluation of copper release and acute local tissue reactions after implantation of copper-coated titanium implants in rats. Biomed. Mater..

[66] Burghardt I., Lüthen F., Prinz C., Kreikemeyer B., Zietz C., Neumann H.G., Rychly J. (2015). A dual function of copper in designing regenerative implants. Biomaterials.

[67] Dawari C.K., Gunell M., Mönkkönen K., Suvanto M., Saarinen J.J. (2022). Antibacterial activity of electrodeposited copper and zinc on metal injection molded (MIM) micropatterned WC-CO hard metals. Coatings.

[68] Van Hengel I., Tierolf M., Valerio V., Minneboo M., Fluit A., Fratila-Apachitei L., Apachitei I., Zadpoor A. (2020). Self-defending additively manufactured bone implants bearing silver and copper nanoparticles. J. Mater. Chem. B.

[69] Popova A.D., Sheveyko A.N., Kuptsov K.A., Advakhova D.Y., Karyagina A.S., Gromov A.V., Krivozubov M.S., Orlova P.A., Volkov A.V., Slukin P.V. (2023). Osteoconductive, osteogenic, and antipathogenic plasma electrolytic oxidation coatings on titanium implants with BMP-2. ACS Appl. Mater. Interfaces.

[70] Augustin A., Thaira H., Udaya Bhat K., Udupa K.R. (2016). Effect of electrodeposited copper thin film on the morphology and cell death of E. coli; an electron microscopic study. Biotechnology and Biochemical Engineering: Select Proceedings of ICACE 2015.

[71] Chen J., He Z., Zheng S., Gao W., Wang Y. (2024). Ultrasonic-assisted electrodeposition of Cu-TiO_2_ nanocomposite coatings with long-term antibacterial activity. ACS Appl. Mater. Interfaces.

[72] Mamleyev E.R., Falk F., Weidler P.G., Heissler S., Wadhwa S., Nassar O., Shyam Kumar C., Kübel C., Wöll C., Islam M. (2020). Polyaramid-based flexible antibacterial coatings fabricated using laser-induced carbonization and copper electroplating. ACS Appl. Mater. Interfaces.

[73] Zeiger M., Solioz M., Edongué H., Arzt E., Schneider A.S. (2014). Surface structure influences contact killing of bacteria by copper. MicrobiologyOpen.

[74] Ciacotich N., Din R.U., Sloth J.J., Møller P., Gram L. (2018). An electroplated copper–silver alloy as antibacterial coating on stainless steel. Surf. Coat. Technol..

[75] Banthia S., Hazra C., Sen R., Das S., Das K. (2019). Electrodeposited functionally graded coating inhibits Gram-positive and Gram-negative bacteria by a lipid peroxidation mediated membrane damage mechanism. Mater. Sci. Eng. C.

[76] Zhang L., Guo J., Yan T., Han Y. (2018). Fibroblast responses and antibacterial activity of Cu and Zn co-doped TiO_2_ for percutaneous implants. Appl. Surf. Sci..

[77] Kharitonov D.S., Kasach A.A., Sergievich D.S., Wrzesińska A., Bobowska I., Darowicki K., Zielinski A., Ryl J., Kurilo I.I. (2021). Ultrasonic-assisted electrodeposition of Cu-Sn-TiO_2_ nanocomposite coatings with enhanced antibacterial activity. Ultrason. Sonochemistry.

[78] Wang X., Ye X., Zhang L., Shao Y., Zhou X., Lu M., Chu C., Xue F., Bai J. (2022). Corrosion and antimicrobial behavior of stainless steel prepared by one-step electrodeposition of silver at the grain boundaries. Surf. Coat. Technol..

[79] El Sayed M.A., Elazab N.T., Gassoumi M., Ibrahim M.A. (2022). Nanocrystalline silver coatings on steel by electrodeposition from non-polluting aqueous baths and its antibacterial activity. J. Taiwan Inst. Chem. Eng..

[80] Kirmanidou Y., Sidira M., Bakopoulou A., Tsouknidas A., Prymak O., Papi R., Choli-Papadopoulou T., Epple M., Michailidis N., Koidis P. (2019). Assessment of cytotoxicity and antibacterial effects of silver nanoparticle-doped titanium alloy surfaces. Dent. Mater..

[81] Reyes-Vidal Y., Suarez-Rojas R., Ruiz C., Torres J., Ţălu Ş., Méndez A., Trejo G. (2015). Electrodeposition, characterization, and antibacterial activity of zinc/silver particle composite coatings. Appl. Surf. Sci..

[82] Zhao Q., Yi L., Jiang L., Ma Y., Lin H., Dong J. (2019). Surface functionalization of titanium with zinc/strontium-doped titanium dioxide microporous coating via microarc oxidation. Nanomed. Nanotechnol. Biol. Med..

[83] Uribe P., Ruiz J., Ortiz C., Blanco S., Gutierrez J. (2020). Antibacterial activity of ZnO nanoparticle coatings formed by electrophoretic deposition. Journal of Physics: Conference Series.

[84] (2025). Sterilization of Health Care Products.

[85] (2022). Geometrical Product Specifications (GPS)—Surface Texture: Profile.

[86] (2021). Biological Evaluation of Medical Devices.

[87] Heidenau F., Mittelmeier W., Detsch R., Haenle M., Stenzel F., Ziegler G., Gollwitzer H. (2005). A novel antibacterial titania coating: Metal ion toxicity and in vitro surface colonization. J. Mater. Sci. Mater. Med..

[88] Haenle M., Fritsche A., Zietz C., Bader R., Heidenau F., Mittelmeier W., Gollwitzer H. (2011). An extended spectrum bactericidal titanium dioxide (TiO_2_) coating for metallic implants: In vitro effectiveness against MRSA and mechanical properties. J. Mater. Sci. Mater. Med..

[89] Gargioni C., Borzenkov M., D’Alfonso L., Sperandeo P., Polissi A., Cucca L., Dacarro G., Grisoli P., Pallavicini P., D’Agostino A. (2020). Self-assembled monolayers of copper sulfide nanoparticles on glass as antibacterial coatings. Nanomaterials.

[90] Fiedler J., Kolitsch A., Kleffner B., Henke D., Stenger S., Brenner R.E. (2011). Copper and silver ion implantation of aluminium oxide-blasted titanium surfaces: Proliferative response of osteoblasts and antibacterial effects. Int. J. Artif. Organs.

[91] Cherenda N.N., Basalai A.V., Shymanski V.I., Uglov V.V., Astashynski V.M., Kuzmitski A.M., Laskovnev A.P., Remnev G.E. (2018). Modification of Ti-6Al-4V alloy element and phase composition by compression plasma flows impact. Surf. Coat. Technol..

[92] Zegan G., Cimpoesu N., Agop M., Stirbu I., Chicet D., Istrate B., Alexandru A., Prisacariu B. (2016). Improving the HA deposition process on ti-based advanced alloy through sandblasting. Optoelectron. Adv. Mater.—Rapid Commun..

[93] Yuda A., Supriadi S., Saragih A. (2019). Surface Modification of Ti-Alloy Based Bone Implant by Sandblasting. AIP Conference Proceedings.

[94] Polishetty A., Manoharan V., Littlefair G., Sonavane C. (2013). Machinability assessment of Ti-6Al-4V for aerospace applications. ASME Early Career Tech. J..

[95] Liang T., Wang Y., Zeng L., Liu Y., Qiao L., Zhang S., Zhao R., Li G., Zhang R., Xiang J. (2020). Copper-doped 3D porous coating developed on Ti-6Al-4V alloys and its in vitro long-term antibacterial ability. Appl. Surf. Sci..

[96] Zhou X., Thompson G.E., Skeldon P., Shimizu K., Habazaki H., Wood G.C. (2005). The valence state of copper in anodic films formed on Al-1at.% Cu alloy. Corrosion.

[97] Whitten J.E. (2023). Ultraviolet photoelectron spectroscopy: Practical aspects and best practices. Appl. Surf. Sci. Adv..

[98] Mansfeldova V., Zlamalova M., Tarabkova H., Janda P., Vorokhta M., Piliai L., Kavan L. (2021). Work function of TiO_2_ (anatase, rutile, and brookite) single crystals: Effects of the environment. J. Phys. Chem. C.

[99] Schultz T., Schlesinger R., Niederhausen J., Henneberger F., Sadofev S., Blumstengel S., Vollmer A., Bussolotti F., Yang J.P., Kera S. (2016). Tuning the work function of GaN with organic molecular acceptors. Phys. Rev. B.

[100] Kashiwaya S., Morasch J., Streibel V., Toupance T., Jaegermann W., Klein A. (2018). The work function of TiO_2_. Surfaces.

[101] Luo Y., Niu L., Wang Y., Wen P., Gong Y., Li C., Xu S. (2023). Regulating the work function of Cu_2_O films via crystal facet engineering with enhanced charge transfer and SERS activity. Appl. Surf. Sci..

[102] Su M., Liang Z., Liu P., Yue S. (2016). Preparation of high quality Cu_2_O crystal and its opto-electronic properties. Mater. Lett..

[103] Kumar S., Datta S., Dey V., Roy D.N. (2025). Formation of nanocones and generation of negative potential on stainless steel surfaces by electrochemical etching synergistically reduce pseudomonas aeruginosa’s biofilm. Surf. Rev. Lett..

[104] Rocha S.S.d., Adabo G.L., Henriques G.E.P., Nóbilo M.A.d.A. (2006). Vickers hardness of cast commercially pure titanium and Ti-6Al-4V alloy submitted to heat treatments. Braz. Dent. J..

[105] Jianchao Y., Wang G., Rong Y. (2015). Experimental study on the surface integrity and chip formation in the micro cutting process. Procedia Manuf..

[106] Chen C.J., Ding S.J., Chen C.C. (2016). Effects of surface conditions of titanium dental implants on bacterial adhesion. Photomed. Laser Surg..

[107] Gallardo-Moreno A.M., Pacha-Olivenza M.A., Saldaña L., Pérez-Giraldo C., Bruque J.M., Vilaboa N., González-Martín M.L. (2009). In vitro biocompatibility and bacterial adhesion of physico-chemically modified Ti_6_Al_4_V surface by means of UV irradiation. Acta Biomater..

[108] Dong Y., Li X., Tian L., Bell T., Sammons R.L., Dong H. (2011). Towards long-lasting antibacterial stainless steel surfaces by combining double glow plasma silvering with active screen plasma nitriding. Acta Biomater..

[109] Sharifahmadian O., Salimijazi H., Fathi M., Mostaghimi J., Pershin L. (2013). Relationship between surface properties and antibacterial behavior of wire arc spray copper coatings. Surf. Coat. Technol..

[110] Cao B., Zheng Y., Xi T., Zhang C., Song W., Burugapalli K., Yang H., Ma Y. (2012). Concentration-dependent cytotoxicity of copper ions on mouse fibroblasts in vitro: Effects of copper ion release from TCu380A vs TCu220C intra-uterine devices. Biomed. Microdevices.

[111] Ruparelia J.P., Chatterjee A.K., Duttagupta S.P., Mukherji S. (2008). Strain specificity in antimicrobial activity of silver and copper nanoparticles. Acta Biomater..

[112] Wennergren D., Bergdahl C., Selse A., Ekelund J., Sundfeldt M., Möller M. (2021). Treatment and re-operation rates in one thousand and three hundred tibial fractures from the Swedish Fracture Register. Eur. J. Orthop. Surg. Traumatol..

[113] Benson D.R., Riggins R.S., Lawrence R.M., Hoeprich P.D., Huston A.C., Harrison J.A. (1983). Treatment of open fractures: A prospective study. J. Trauma Acute Care Surg..

[114] Prinz C., Elhensheri M., Rychly J., Neumann H.G. (2017). Antimicrobial and bone-forming activity of a copper coated implant in a rabbit model. J. Biomater. Appl..

[115] Hill P., Clasper J., Parker S., Watkins P. (2002). Early intramedullary nailing in an animal model of a heavily contaminated fracture of the tibia. J. Orthop. Res..

[116] Moriarty T., Schmid T., Post V., Samara E., Kates S., Schwarz E., Zeiter S., Richards R. (2017). A large animal model for a failed two-stage revision of intramedullary nail-related infection by methicillin-resistant *Staphylococcus aureus*. Eur. Cell. Mater..

[117] Banstola A., Reynolds J.N.J. (2022). The Sheep as a Large Animal Model for the Investigation and Treatment of Human Disorders. Biology.

