# Supplementary material for: In Vitro Antibacterial Efficacy of a New TiO2-Cu-Coated Titanium Surface for Biomedical Applications
Source: Nanomaterials (Basel). 2025 Nov 19;15(22):1742. doi: 10.3390/nano15221742 (PMC12655840; doi:10.3390/nano15221742)
Supplement: Supplementary file 1 [file nanomaterials-15-01742-s001.zip › nanomaterials-3859438-supplementary.pdf]

Table S1: Elemental composition of the surface of C-D and Cu-D samples upon analysis with EDS and ICP-OES. ∅ indicates that the element was not detected.

		EDS		ICP-OES
	Element	Atomic concentration (%)	Weight concentration (%)	Concentration (mg/ml)
C-D	O	58.8	35.7	∅
	Al	4.8	4.9	0.61
	Si	9.2	9.8	1.51
	Ti	25.7	47.0	9.97
	V	1.3	2.6	0.71
	**Cu**	**∅**	**∅**	**∅**
	Fe	∅	∅	0.091
	Ca	∅	∅	0.0047
	Na	∅	∅	0.10
Cu-D	O	51.2	27.4	∅
	Al	4.0	3.6	0.50
	Si	13.3	12.5	1.70
	Ti	19.5	31.2	8.18
	V	0.9	1.6	0.73
	**Cu**	**10.9**	**23.1**	**12.64**
	Fe	∅	∅	0.11
	Ca	∅	∅	0.085
	Na	∅	∅	0.16

Table S2: Elemental composition of C-D and Cu-D obtained with XPS analysis. ∅ indicates that the element was not detected.

	**Elements**	**O 1s**	**C 1s**	**Na 1s**	**Ti 2p**	**Si 2p**	**Cu 2p**	**Al 2p**
**Atomic conc. (%)**	*C-D*	50.5±0.5	22.5±0.2	1.5±0.1	5.5±0.2	19.9±0.3	∅	∅
	Cu-D	32.9±0.5	31.5±1.5	1.0±0.1	1.5±0.1	12.5±0.2	1.1±0.2	1.9±1.1

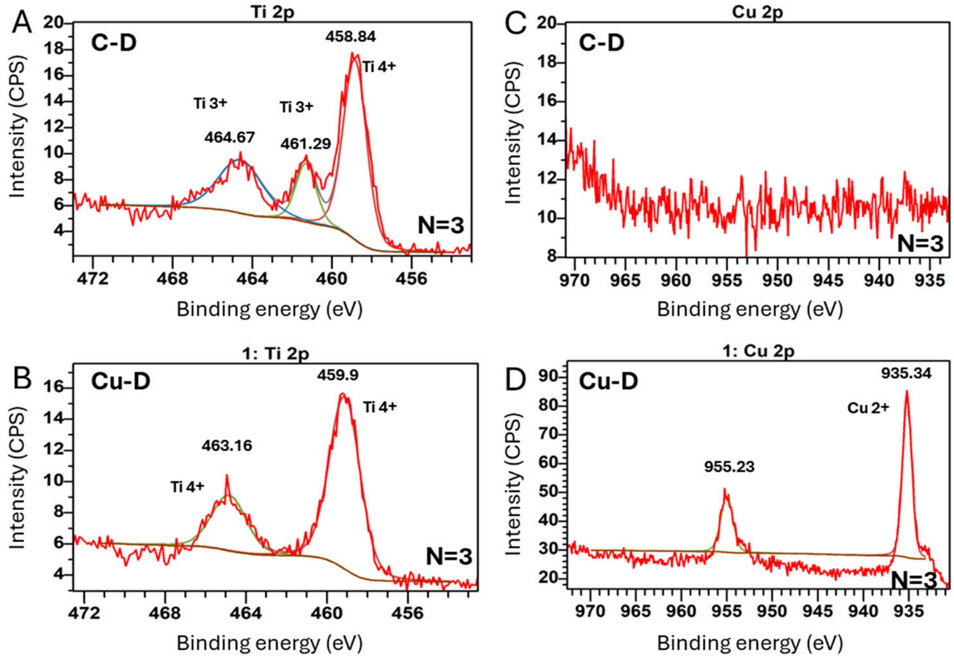

Figure S1: Detailed XPS results including: (A) High-resolution spectrum of Ti 2p for C-D group, (B) High-resolution spectrum of Cu 2p for C-D group, (C) High-resolution spectrum of Ti 2p for Cu-D group, and (D) High-resolution spectrum of Cu 2p for Cu-D group.

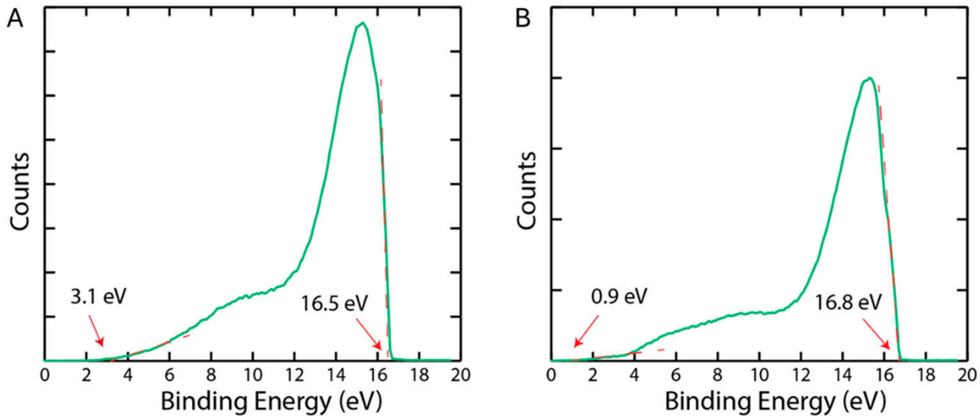

Figure S2: UPS spectra of A) Ti samples and B) Cu-Ti sample, showing the determination of secondary electron cutoff (SECO) and valance band maximum (VBM), from which surface band bending potential was calculated.
